# Long‐term follow‐up of a randomised controlled trial of a brief home‐based parenting intervention to reduce behavioural problems in young children

**DOI:** 10.1111/jcpp.70037

**Published:** 2025-09-17

**Authors:** Paul Ramchandani, Jack Elkes, Victoria Cornelius, Sarah Byford, Laura Oxley, Daphne Babalis, Beth Barker, Erin Bibby, Brittney Chere, Poushali Ganguli, Sam Griffith, Zaheema Iqbal, Aiman Kamarudin, Katie Lui, Stephen Scott, Emma Tassie, Essi Viding, Christine O'Farrelly

**Affiliations:** ^1^ University of Cambridge Cambridge UK; ^2^ Imperial College London London UK; ^3^ King's College London London UK; ^4^ University College London London UK

**Keywords:** Early intervention, behavioural problems, RCT design, longitudinal studies

## Abstract

**Background:**

Behaviour problems are common in childhood and are associated with higher rates of mental health problems, educational and relationship difficulties throughout life. This study assessed whether a Video‐feedback Intervention to promote Positive Parenting and Sensitive Discipline (VIPP‐SD) has sustained benefit 6 years after delivery. It had previously been shown to reduce behavioural problems in children aged 2 and 4 years old.

**Methods:**

The Healthy Start, Happy Start study was a 2‐arm, multisite randomised clinical trial conducted in 6 NHS trusts in England. Participants (*N* = 300) were parents/caregivers of children (aged 12–36 months) at risk of behaviour problems. Participants were randomised to receive either VIPP‐SD (*n* = 151) or usual care (*n* = 149). Those allocated to VIPP‐SD were offered 6 home‐based video‐feedback sessions. Six‐year follow‐up data were collected from May 2022 to July 2023. The primary outcome was the total score on Parental Account of Children's Symptoms (PACS). The analysis used prespecified longitudinal Bayesian models to handle missing data, and findings are reported as posterior probabilities of superiority alongside treatment effect estimates with 95% credible interval.

**Results:**

Analysis included 294 of the 300 participants, with 6‐year primary outcome data available for 244/300 (81%) (106 girls [43%]; mean age, 8.2 years). The probability of superiority for VIPP‐SD on PACS was 86%. The mean difference in the total PACS score was −1.23 (95% Cred.I [−3.34, 0.90]); *d* = −0.11 (95% Cred.I [−0.032, 0.09]), with fewer behavioural problems in children in the VIPP‐SD group (mean [*SD*] score of 25.30 [9.63] vs. 26.36 [11.05]).

**Conclusions:**

This trial found a probability of 86% that VIPP‐SD was superior for reducing behaviour problems in children up to 6 years later. Taken together with the earlier positive trial findings, this suggests a small enduring positive impact of a brief early intervention with potential for scaling.

## Background

A growing number of children and young people, as many as one in five, have a probable mental health disorder (Newlove‐Delgado et al., 2023). These often begin in early childhood, with behaviour problems being the commonest and having one of the earliest onsets (Cree et al., 2018; von Klitzing, Döhnert, Kroll, & Grube, 2015; Wakschlag et al., 2019). Where behaviour problems endure, they place children at increased risk of mental health disorders, antisocial behaviour, drug and alcohol misuse, poorer educational attainment and physical ill health into adulthood (Ramchandani et al., 2017). Thus, early behavioural problems significantly burden children, families and society during early childhood and into children's futures.

Early intervention is increasingly situated as a key, sustainable way of tackling the childhood mental health crisis as it capitalises on greater brain plasticity and can intercept problems before they become established (Doyle, Harmon, Heckman, & Tremblay, 2009). This position forms the basis of early intervention policies, as it alleviates distress for children and families as early as possible and may offer substantial long‐term returns on investment for health, education, criminal justice and social welfare systems (Skokauskas et al., 2018). Indeed, parenting interventions are considered likely to be a cost‐effective use of societal resources in terms of prevention of child externalising and internalising behaviours (Sampaio, Nystrand, Feldman, & Mihalopoulos, 2024).

Yet few studies have adequately tested whether treatment effects and cost savings are amplified when preventive interventions are delivered in early childhood, whether benefits are sustained over time and the extent to which children may need additional later support (Maughan & Barker, 2019; Moffitt & Scott, 2008). A significant impediment is the lack of follow‐up studies, particularly of very early intervention; meta‐analyses show that relatively few studies commence in the first 3 years of childhood, and follow‐up periods beyond 2 years are rare (Kaminski, Claussen, Sims, & Bhupalam, 2024; Mingebach, Kamp‐Becker, Christiansen, & Weber, 2018; van Aar, Leijten, de Castro, & Overbeek, 2017). This is also the case for economic evaluations, which have typically relied on short‐term horizons below 1 year, have limited costing perspectives and face challenges with outcome assessment (Sampaio et al., 2024).

In our two‐group, researcher‐blind, multisite pragmatic randomised controlled trial, 300 families of young children (aged 12–36 months) at risk of behaviour problems were randomised to receive a brief parenting intervention (Video‐feedback Intervention to promote Positive Parenting and Sensitive Discipline; VIPP‐SD) and usual care or usual care (UC) only. VIPP‐SD targets parents' sensitivity and sensitive discipline and is suitable for children as young as 12 months. We previously observed a positive postintervention treatment effect on a structured interview of children's behaviour (standardised effect size *d* = 0.20) that was largely sustained at a 2‐year postrandomisation follow‐up (*d* = 0.17). This study aimed to examine the effectiveness and cost‐effectiveness of VIPP‐SD at a longer term follow‐up, 6 years postrandomisation, when children were aged 8 years.

## Methods

### Study design and participants

This study is a follow‐up at 6 years postrandomisation of a two‐arm (1:1), parallel group, multisite, pragmatic randomised controlled trial (see O'Farrelly et al., 2021, for details of recruitment and randomisation process). We compared the use of a brief parenting intervention (VIPP‐SD) to usual care for parents/carers of young children (aged 12–36 months) at risk of behaviour difficulties (those children scoring in the top 20% (≥8 on the externalising subscale)) on the Strengths and Difficulties Questionnaire (SDQ) (Goodman, 1997). VIPP‐SD was predominantly delivered through NHS health visiting teams.

Children were aged between 6 and 9 years (average 8 years) at the time of the follow‐up study assessment. The study used well‐established interview and questionnaire measures to assess child outcomes, focussing on child behaviour.

Ethics approval for the study was given by the London‐Surrey NHS Research Ethics Committee (22/LO/0182). The study was registered with the Integrated Research Approval System (IRAS) under the reference number 310487. The sponsor for the study was the University of Cambridge.

Participants in this follow‐up study needed to meet the following eligibility criteria.

#### Inclusion criteria

1. Family participated in the original Healthy Start, Happy Start trial.
2. Written informed parental consent from participating caregivers.

#### Exclusion criteria

1. Child or parent has severe sensory impairment, learning disability or language limitation that is sufficient to preclude participation in the study.

### Randomisation and masking

Participants were randomised to the intervention group (VIPP‐SD) or Usual Care (UC) at the beginning of the original Healthy Start Happy Start trial. The randomisation procedure is described in detail elsewhere (O'Farrelly, Barker, et al., 2021). Researchers assessing the study outcomes in this 6‐year follow‐up study were blinded to group allocation of participants. The statistician was unblinded for analysis.

### Procedures

#### Setting

Families were recruited for the original trial from NHS healthcare settings in London (Camden, Hillingdon, Islington, and Barking and Dagenham), Peterborough, Oxfordshire and Hertfordshire between July 2015 and July 2017. The original recruitment was primarily conducted through health visiting services.

#### Recruitment procedure

A total of 300 families (child aged 12–36 months, and one or two participating caregivers) were recruited into the original Healthy Start, Happy Start trial between July 2015 and July 2017. All participants in the original trial consented to their data being stored and used to contact them again for further related research. Participating caregivers provided written, electronic informed consent.

Although consent for the children's participation was provided by their caregiver, participating children were also invited to take part in a child assent procedure. A Child Information Booklet was presented in a format designed to be easily understood by young children.

#### Data collection

The primary end‐point measure (Parental Account of Childhood Symptoms (PACS) interview) was carried out via telephone or video call with the caregiver. The interview was audio recorded. Following completion of the telephone interview, a home visit was scheduled with families to collect secondary outcome data. These visits were predominantly carried out in the participants' homes. For participants who had moved too far away for a home visit to be feasible in person (e.g. outside the UK), this assessment was conducted remotely via video call.

Family demographic information was collected, including the caregiver's gender, age, ethnicity, education level, employment status and relationship status; and the child's age, sex and ethnicity.

The telephone interview took around 1 hr and 15 min to complete. The home visit took around 2 hr to complete. All telephone interviews and home visits were conducted between May 2022 and July 2023.

### Intervention

This study used the VIPP‐SD intervention (Juffer, BakermansKranenburg, & van IJzendoorn, 2008; Juffer, Bakermans‐Kranenburg, & van IJzendoorn, 2017; van IJzendoorn, Schuengel, Wang, & Bakermans‐Kranenburg, 2023) which was delivered to parents/caregivers by trained therapists over six sessions (each ranging from 1 to 2 hr long) during home visits on a fortnightly basis. Trained therapists, predominantly health visitors, community nursery nurses and psychologists, delivered the VIPP‐SD intervention.

During the first half of the sessions, therapists filmed interactions between the parent and child, which were then later analysed by the therapist in preparation for providing feedback at the next session. In the second half of the session, therapists provided parents with individualised feedback from the previous session.

### Usual care

Participants in both groups (intervention and usual care) continued to receive their usual care.

### Outcome measures

Unless otherwise stated, all measures were completed by the child's primary caregiver.

#### Primary outcome

##### Parental Account of Childhood Symptoms

The Parental Account of Childhood Symptoms (PACS) is a semi‐structured interview administered by trained researchers and conducted with the child's primary caregiver. It is used for the assessment of children aged over 5 years old (Taylor et al., 1986). At previous timepoints in the trial, we had used the Pre‐PACS, a version of the PACS adapted for preschool children (aged under 5 years).

During the PACS interview, the caregiver is asked to recall and describe detailed examples of their child's typical behaviour over the past week in a range of settings (e.g. in the home, with friends, in public) to capture behaviour based on real examples. To ensure that the example given is characteristic of the child, caregivers were asked how representative the described behaviour is of the child over the past 4 months. The researcher rates the severity and frequency of the child's symptoms, guided by written definitions and thresholds of each of the scored behaviours. The interview is used to score the children on two subscales: ADHD/hyperkinesis and conduct problems.

This measure has high inter‐rater reliability, good construct validity and has been used as an outcome measure in a number of other clinical trials assessing intervention effects on child behaviour (Keown, 2012; Taylor et al., 1986).

#### Secondary outcomes

##### Child Behaviour Checklist

Behaviour problems were measured using the Child Behaviour Checklist (CBCL 5–18 years) (Achenbach, 1999). The CBCL is a well‐validated and widely used 113‐item questionnaire that asks parents to rate how true the behaviour is of their child over the last 6 months on a 3‐point scale (0 = *not true*, 1 = *somewhat true* or 2 = *very true or often true*).

##### Strengths and Difficulties Questionnaire

The Strengths and Difficulties Questionnaire (SDQ) is a robust and reliable measure of child behaviour (Goodman, 1997). The questionnaire consists of 25 items that make up 5 subscales. Each question asks about a specific behaviour and is rated; 0 = *not true*, 1 = *somewhat true* or 2 = *certainly true*. The subscale scores (not including the prosocial behaviour subscale) can be combined to generate an overall difficulties score (range 0–40). Where parental consent for contact with the child's school was given, the child's schoolteacher was also invited to complete the SDQ.

##### Callous‐Unemotional Traits Scale

Children's Callous‐Unemotional traits (CU traits) were assessed using four items from the SDQ prosocial scale (reverse scored) and three items from the Inventory of Callous‐Unemotional Traits (ICU), combined into a scale, in line with the short CU trait assessment used by other groups (Dadds et al., 2014; Takahashi, Pease, Pingault, & Viding, 2021).

### Economic measures

#### Child Health Utility‐9D

The Child Health Utility‐9 Dimensions (CHU9D) is a preference‐based, generic measure of health‐related quality of life capable of generating Quality‐Adjusted Life Years (QALYs). This questionnaire was used to measure effectiveness for the economic evaluation. The CHU9D was self‐completed by the children. QALYs were estimated directly from the CHU9D (Furber & Segal, 2015; Stevens, 2012). Full details are provided in the Appendix Section S2.

#### Child and Adolescent Service Use Schedule

Information on the use of health and social care services was recorded using a modified version of the Child and Adolescent Service Use Schedule (CA‐SUS), completed in interview with the child's caregiver. Respondents are asked to recall use (number of appointments/sessions/nights etc.) of a range of services, including accommodation, hospital and community health and social care services, and prescribed medication. Given the long follow‐up period since the end of the original RCT, two versions of the CA‐SUS were developed. First, a comprehensive version covering the same services as collected in the original RCT, which focused on the 3‐month period prior to the 6‐year follow‐up interview (‘3‐month CA‐SUS’) and a briefer version focused on key and more easily recalled resources (high cost and/or high use) covering the full period since the last interview (‘3‐year CA‐SUS’). Further details are provided in Section S2 of the appendix.

#### Other secondary outcome measures

Four other parental questionnaire measures were collected (PHQ‐9 for depression; GAD‐7 for anxiety; RDAS for couple relationship and BPSES for parenting self‐efficacy). These are described in more detail in Section S1 of the appendix.

### Statistical analysis

#### Data management

See Appendix S1.

#### Data analysis

All statistical analysis was performed using Stata 17.

A Bayesian framework for the analysis was prespecified in our application for funding, as well as in other trial documentation (e.g. protocol and SAP). The decision to use this approach (rather than a frequentist one) was made because the sample size was fixed from the original trial (*N* = 300), and given the anticipated loss to follow‐up of some participants over this longer follow‐up period, the follow‐up study would not have sufficient power to perform hypothesis testing. The final statistical analysis plan was reviewed by the Project Management Group (PMG) and Study Steering Committee (SSC) and signed off prior to data extraction for analysis.

The primary end‐point was the difference in PACS scores at 6 years. Pre‐PACS scores were standardised against the overall mean at baseline, and PACS scores were standardised against the mean at 6 years in the control arm. Standardised scores were analysed using a Bayesian longitudinal linear model that assumes missing data to be missing at random. The models included the intervention arm and an interaction between the intervention arm and postbaseline timepoint with adjustment for the standardised baseline Pre‐PACS total score, baseline age of the child and original stratification variables, number of caregivers in the trial and site. Vague priors were fitted to all fixed components of the model, with a mean of 0 and a standard deviation of 100. To evaluate the potential strength of evidence the follow‐up study could provide, based on differing sample sizes due to uncertain loss to follow‐up, we undertook simulations on three potential scenarios (see Appendix S1 and Table S1 within, for further information). The same modelling approach was used for all secondary outcomes except for BPSES and CUTS, which were only captured at the 6‐year follow‐up visit. Here, a linear regression model was used.

The primary estimand targeted a treatment policy research question with the intention to treat population who attended at least one postbaseline assessment. Three supplementary estimands used a principal stratum approach to determine the intervention effect in participants who attended 4/6 sessions of VIPP‐SD; carers had not attended any alternative parenting interventions; and had a confirmed neuro‐developmental diagnosis (see Appendix Section S3, for further information). Supplementary analysis using two‐stage least‐squares regression in a frequentist framework; no *p*‐values are reported.

A sensitivity analysis assessed the impact of missing data on the primary outcome using a delta‐based multiple imputation. Imputations were estimated using primary analysis model covariates and auxiliary variables (CBCL, SDQ and the three intercurrent events). Delta was 2.03, the intervention effect at 24 months (O'Farrelly et al., 2021). Five sensitivity analyses were performed: recovery of participants without postbaseline data (imputations not shifted), all imputed shifted lower (i.e. did worse), control arm shifted up (i.e. did better), control arm down and finally VIPP‐SD arm shifted down.

### Health economic analysis

The economic evaluation took a health and social care perspective, in line with the perspective in the HSHS RCT (O'Farrelly, Barker, et al., 2021). Nationally relevant unit costs were applied to all service use (detailed in the Appendix S2), with the exception of the HSHS intervention, which was directly costed in the original HSHS RCT (O'Farrelly, Watt, et al., 2021). The base case primary economic analysis was a cost‐utility analysis using QALYs as the measure of effect and with costs estimated from the 3‐month CA‐SUS. A secondary cost‐effectiveness analysis was carried out using the PACS, and sensitivity analyses explored the impact of missing data using multiple imputation with chained equations and the impact of using costs estimated from the 3‐year CA‐SUS (see Appendix S2).

Differences in costs and outcomes were estimated using linear regression models. Nonparametric bootstrapping with 5,000 estimates was used to estimate the 95% confidence intervals around mean differences to account for the non‐normal distribution associated with economic data (Barber & Thompson, 2000). Cost‐effectiveness of VIPP‐SD compared with usual care was explored using a Bayesian framework (Briggs, 1999) by calculating incremental cost‐effectiveness ratios (ICERs) (the difference in cost, divided by the difference in outcome) (Van Hout, Al, Gordon, & Rutten, 1994). To characterise the uncertainty surrounding the estimates of incremental cost‐effectiveness, bootstrapping was used to generate 10,000 estimates of mean costs and effects by trial arm, which were plotted on a cost‐effectiveness plane. Cost‐effectiveness acceptability curves (CEACs) were constructed using the net monetary benefit approach to examine the probability of VIPP‐SD being cost‐effective compared with usual care for a range of willingness to pay (WTP) thresholds per unit of improvement (Fenwick & Byford, 2005; Stinnett & Mullahy, 1998). In line with the clinical analysis, all economic analyses were adjusted for recruitment centre, age of the child at follow‐up and number of parents/carers participating, as well as baseline values of the variables of interest (e.g. baseline costs and effects).

## Results

In the initial trial, 300 parents/carers of children (aged 12–36 months) were allocated to receive either VIPP‐SD and usual care or usual care (UC) only. Findings from the postintervention and 2‐year follow‐up have been previously reported (O'Farrelly, Barker, et al., 2021; O'Farrelly, Watt, et al., 2021) demonstrating a sustained effect of the intervention on children's behavioural problems.

**Figure 1:**
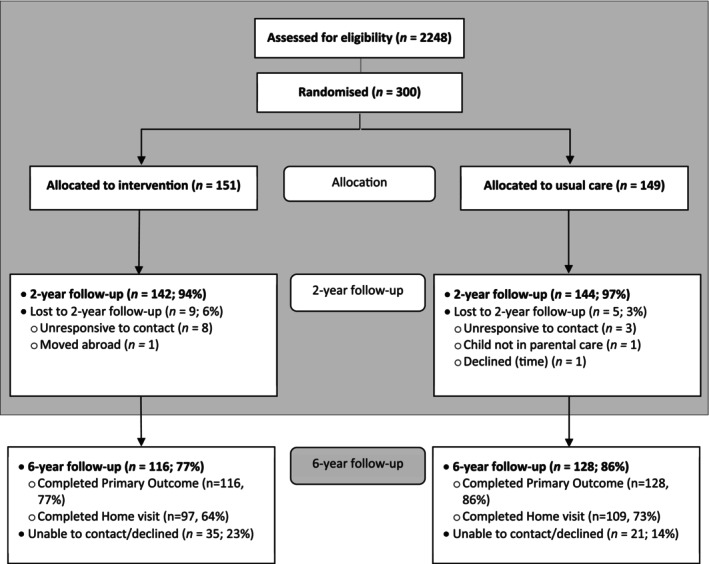
The CONSORT flow diagram for the Healthy Start, Happy Start randomised controlled trial. Grey area has already been completed as part of the original HSHS trial (HTA: 13/04/33)

The retention rate for this 6‐year follow‐up study was 81% (*N* = 244/300). Children had a mean age of 8 years at the time of this study. Participant characteristics are shown in Table 1. The figure shows participant flow (Figure 1).

**Table 1:** Comparison of demographics for primary caregivers between those in the original trial at baseline and those in the follow‐up study

Demographic characteristic	Intervention							
	Control				VIPP‐SD			
	Original (*N* = 149)		Follow‐up (*N* = 128)		Original (*N* = 151)		Follow‐up (*N* = 116)	
Caregiver age, mean (*SD*)	34.7	(5.89)	41.3	(5.21)	33.7	(5.58)	41.8	(5.18)
		***n* (%)**		***n* (%)**		***n* (%)**		***n* (%)**
Proportion female primary caregivers	144	(96.6)	113	(96.6)	143	(94.7)	99	(94.3)
Relationship to child of primary caregiver								
Biological mother	143	(96.0)	105	(96.3)	144	(95.4)	92	(93.9)
Biological father	4	(2.7)	3	(2.8)	7	(4.6)	6	(6.1)
Adoptive mother	1	(0.7)	1	(0.9)				
Adoptive father	1	(0.7)						
Ethnicity of primary caregiver								
Asian or Asian British	16	(10.7)	13	(11.9)	15	(9.9)	10	(10.2)
Black, Black British, Caribbean or African	15	(10.1)	11	(10.1)	3	(2.0)	4	(4.1)
Mixed or multiple ethnic groups	11	(7.4)	7	(6.4)	11	(7.3)	8	(8.2)
White	103	(69.1)	71	(65.1)	114	(75.5)	72	(73.5)
Other ethnic group	4	(2.7)	7	(6.4)	8	(5.3)	4	(4.1)
Relationship status								
Legally separated	1	(0.7)	1	(0.9)	1	(0.7)	1	(1.0)
Married	98	(65.8)	75	(68.8)	84	(55.6)	63	(64.3)
Single never married and never in a civil partnership	17	(11.4)	12	(11.0)	12	(7.9)	13	(13.3)
Cohabiting	29	(19.5)	18	(16.5)	42	(27.8)	15	(15.3)
Divorced	2	(1.3)	3	(2.8)			3	(3.1)
Widowed	1	(0.7)						
A civil partner in a legally recognised civil partnership					2	(1.3)	1	(1.0)
In a relationship but not cohabiting	1	(0.7)			10	(6.6)	2	(2.0)
Highest qualification								
Pre‐GCSE	3	(2.0)	1	(0.9)	6	(4.0)	1	(1.0)
GCSEs	11	(7.4)	10	(9.3)	11	(7.3)	5	(5.2)
College, i.e. A levels, NVQ, BTEC	36	(24.2)	22	(20.6)	42	(27.8)	20	(20.8)
Undergraduate	43	(28.9)	28	(26.2)	35	(23.2)	19	(19.8)
Postgraduate	56	(37.6)	46	(43.0)	57	(37.7)	51	(53.1)
Employment status								
Working for an employer	64	(43.0)	61	(56.0)	66	(43.7)	74	(75.5)
Paid maternity/paternity/parental leave from an employer	10	(6.7)			6	(4.0)	1	(1.0)
Self‐employed	12	(8.1)	14	(12.8)	20	(13.2)	10	(10.2)
Full‐time student	7	(4.7)	6	(5.5)	3	(2.0)	1	(1.0)
Looking after the home and family	56	(37.6)	28	(25.7)	56	(37.1)	12	(12.2)

Primary outcome (child behaviour on PACS) results are given in Table 2. At 6‐year follow‐up, there was a high probability (86%) for superiority of VIPP‐SD on the PACS score with an adjusted between arm difference in mean scores of 1.23 (95% Cred.I [−3.34, 0.90]); *d* = −0.11 (95% Cred.I [−0.032, 0.09]). There were fewer behavioural problems in children in the VIPP‐SD group (unadjusted mean [*SD*] score of 25.30 [9.63]), than children in the control group (mean [*SD*] 26.36 [11.05]). In those that received 4 or more sessions of VIPP‐SD, the estimated adjusted standardised effect was larger at −0.14 (95% confidence interval [−0.39 to 0.10]).

**Table 2:** Primary analysis of PACS scores over time and at the last follow‐up only and in each subdomain separately

Outcome	Period assessed	*N*	Intervention effect estimate	95% Credible interval	Posterior probability of superiority (MCSE)
**Standardised total score (Primary)**	**All timepoints**	**294**	**−0.11**	**−0.32 to 0.09**	**0.86 (0.00)**
Totals scores	All timepoints	294	−1.23	−3.34 to 0.90	0.87 (0.00)
Standardised behaviour total score	All timepoints	294	−0.12	−0.31 to 0.08	0.88 (0.00)
Behaviour total score	All timepoints	294	−0.86	−2.18 to 0.45	0.90 (0.30)
Standardised hyperactivity total scores	All timepoints	294	−0.06	−0.27 to 0.16	0.70 (0.00)
Hyperactivity total score	All timepoints	294	−0.36	−1.69 to 0.96	0.70 (0.00)

MCSE, Monte‐Carlo Standard Error.

Analysis of the two components of the PACS (behaviour score and hyperactivity score) showed that the VIPP‐SD intervention effect was driven more by the effect on the behaviour subdomain (*d* = −0.12, 95% CI [−0.31 0.08] PPS 88%), than the hyperactivity subdomain (*d* = −0.06, 95% CI [−0.27 0.16] PPS 70%), consistent with findings at earlier timepoints in the trial.

Additional sensitivity analysis conducted on the primary outcome (PACS score) using all participants gave a standardised effect size estimate of −0.19 (95% CI [−0.41 0.03]) with a posterior probability of superiority of 0.95. Extensive sensitivity analysis for missing data resulted in an estimated standardised effect size from −0.15 to −0.21 with a posterior probability of superiority from 0.91 to 0.97 (see Appendix S1, Table S2). These point to a higher estimate of effect than our stated primary outcome when missing participant data were included.

The mean group differences in primary caregiver‐reported CBCL and SDQ scores were also consistent with the primary outcome findings, with a probability of superiority for VIPP‐SD of 82% and 79%, respectively, favouring the intervention group (Tables 3 and 4).

**Table 3:** Descriptive summary of mean secondary outcome scores by intervention arm at 6‐year follow‐up timepoint

Secondary outcome	Intervention			
	Control		VIPP‐SD	
	(*N* = 128)		(*N* = 116)	
	*N*	Mean (*SD*)	*N*	Mean (*SD*)
CBCL	113	31.97 (23.554)	102	26.05 (20.496)
Parent – SDQ	113	11.25 (6.494)	102	10.08 (5.644)
Teacher – SDQ	55	7.31 (6.591)	46	8.91 (6.742)
PHQ9	113	4.70 (5.053)	102	3.96 (4.441)
GAD7	113	4.81 (4.418)	102	4.34 (4.055)
RDAS	98	48.84 (8.715)	85	49.95 (8.304)
CUTS	108	3.49 (2.370)	102	2.98 (2.129)
BPSES	113	21.42 (2.812)	102	20.35 (4.286)

BPSES, Brief Parental Self‐Efficacy Scale; CBCL, Child Behaviour CheckList; CUTS, Callous‐Unemotional Traits Scale; GAD7, Generalised Anxiety Disorder; PHQ9, Patient Health Questionnaire; RDAS, Revised Dyadic Adjustment Scale; SDQ, Strengths and Difficulties Questionnaire.

**Table 4:** Estimated intervention effect of secondary outcomes over time and also at the 6‐year timepoint

Secondary outcome	Intervention effect estimate	Period assessed	95% Credible interval	Posterior probability of superiority
CBCL total score	−2.12	All timepoints	−6.66 to 2.40	0.82
Parent SDQ total score	−0.50	All timepoints	−1.75 to 0.76	0.79
PHQ‐9 total score	−0.50	All timepoints	−1.49 to 0.50	0.84
GAD‐7 total score	−0.38	All timepoints	−1.33 to 0.58	0.78
RDAS total score	−0.16	All timepoints	−2.00 to 1.67	0.56
CUTS total score^a^	−0.60	6‐year follow‐up	−1.21 to 0.03	0.97
BPSE scale^a^	−1.00	6‐year follow‐up	−1.99 to −0.03	0.02

^a^ These are reported only with end‐point data (as they were not collected at earlier timepoints).

There was also moderately strong evidence of group differences in all the other secondary outcomes (parent or caregiver‐reported mood, anxiety, severe child behaviours), but no evidence of any difference for couple functioning (Tables 3 and 4).

Service use, mean costs per participant, QALYs and PACS scores for participants with full economic data are discussed and presented in detail in the Appendix S2 (Tables S3–S6). In summary, accommodation, hospital, and community health and social care costs were all lower in the VIPP‐SD group compared with usual care. However, total costs were higher for VIPP‐SD when the cost of the intervention was included (adjusted mean difference £1,479, 95% CI −380 to 3,338). In terms of outcomes, QALYs were higher (better health‐related quality of life), and PACS scores were lower (fewer behavioural problems) in the VIPP‐SD group. Table 5 summarises the base case economic analysis and sensitivity analyses. In the base case – a complete case cost‐utility analysis with QALYs as the measure of effect and using costs estimated using the 3‐month CA‐SUS – both costs and QALYs were higher in the VIPP‐SD group compared with usual care (higher costs, better outcomes). The ICER of £33,607 per QALY falls above the £20,000–£30,000 cost per QALY threshold preferred by NICE (2023), with probabilities of VIPP‐SD being cost‐effective compared with usual care at these thresholds being 30% and 46%, respectively. VIPP‐SD remained cost‐ineffective compared with usual care when missing data were imputed (ICER £56,487; probabilities 20% and 29%), but when using costs estimated using the 3‐year CA‐SUS, the ICER fell below the £30,000 per QALY threshold (£26,168). This gave a probability of VIPP‐SD being cost‐effective of 60%. Secondary analysis using the PACS suggests that society would need to be willing to pay at least £582 per unit improvement in PACS score for VIPP‐SD to have a higher probability of being cost‐effective than usual care. Associated cost‐effectiveness planes and cost‐effectiveness acceptability curves are presented in Appendix S2, Figures S1–S8.

**Table 5:** Base case, sensitivity and secondary economic analyses

	Mean difference in costs^a^	Mean difference in effects^a^	ICER^b^	Probability VIPP‐SD cost‐effective compared with usual care^c^
Base case (QALYs; 3‐month CA‐SUS)	1,479	0.044	£33,607	30% to 46%
Base case with missing data imputed^d^	1,638	0.029	£56,487	20% to 29%
Base case with 3‐year CA‐SUS	1,151	0.044	£26,168	36% to 60%
Secondary (PACS)	1,479	−2.54	£582	n/a

^a^ Adjusted for recruitment centre, age of child at follow‐up, number of caregivers participating and baseline values.

^b^ Incremental cost‐effectiveness ratio.

^c^ Based on a willingness to pay for a QALY between £20,000 and £30,000.

^d^ Multiple imputation based on 1,000 bootstrap iterations.

### Adverse events

No adverse events were reported in this study.

## Discussion

In this study, we found a probability of 86% of a continued beneficial effect of the VIPP‐SD intervention 6 years after the initial delivery of this brief six‐session intervention. These findings, taken together with the positive findings from the original trial, suggest a sustained, though diminished, long‐term effect on children's behaviour.

The finding of a smaller effect size (ES) for the VIPP‐SD intervention (0.11) at 6‐year follow‐up compared with the immediate post‐treatment ES of 0.2 is probably to be expected, given the significant additional time that has passed. Although a standard effect size of 0.11 can be difficult to interpret for an individual child, the rated mean difference between the groups of 1.23 points on the PACS measure does represent a noticeable difference in child behaviour. As an example, a 1‐point change on the tantrums scale would represent a change from marked (throwing things and kicking objects) to mild (waving arms or stamping feet). These kinds of effects are likely to be experienced as a very meaningful difference to a child and their classmates, teachers and parents' experiences of daily life at home and school. The value of intervention should be assessed based on all the evidence, and it is important to note that the effect size of 0.11 seen here at the 6‐year follow‐up should be considered along with the earlier findings of a postintervention effect size of 0.2 and an effect size of 0.17 at the 2‐year follow‐up. Making a difference early in children's lives can be important for them and their families, even if the measured effects diminish somewhat over time.

It is of note that changes are predominantly seen in child behavioural disturbances such as aggression and other conduct scores, rather than hyperactivity (an effect size of −0.12 for conduct vs. −0.06 for hyperactivity). This is broadly consistent with the sensitive discipline focus of the intervention, which targets conduct problems. Meta‐analyses on other parenting interventions also show that effect sizes are higher for independently rated conduct problems than independently rated ADHD symptoms (Daley et al., 2014; Rimestad, Lambek, Zacher Christiansen, & Hougaard, 2019). Similarly, Overbeek et al. (2021) found sustained effects of the incredible years on parent‐reported conduct problems, but not on parent‐reported ADHD symptoms, at 2.5‐year follow‐up. They posit that ADHD symptoms may be less driven or maintained by parenting behaviour (Burke, Pardini, & Loeber, 2008) and thus require programmes more specialised to ADHD.

The economic findings were mixed. Whilst the cost of hospital and community health and social care services were lower in the VIPP‐SD group, this was not enough to offset the additional cost of the VIPP‐SD intervention. The collection of accurate service use data over such a long period of time is difficult, and results varied dependent on the approach taken. In comparison with other aspects of economic evaluation, such as the measurement of health outcomes, there is little guidance in the literature relating to the accurate measurement of resource use. However, some evidence suggests that participants may have better recall with certain types of service use, for example more salient episodes such as overnight hospital stays (Stull, Leidy, Parasuraman, & Chassany, 2009). We, therefore, took two approaches – the collection of detailed data over a short period of time (3 months) and the collection of data on a smaller number of key services (high cost and/or high use) over the full follow‐up period. The former is likely to be more accurate, but the process of multiplying up to cover the full follow‐up period introduces errors. The latter requires respondents to recall over a significant length of time and thus is likely to contain reporting errors, but no further errors are introduced via multiplication. These alternative approaches to service use measurement yielded a mixed picture, with VIPP‐SD being cost‐ineffective compared with usual care using data collected over 3 months but cost‐effective at the £30,000 per QALY threshold when using data collected over the full follow‐up period.

Before considering the important implications of these findings, there are several strengths and limitations to review. First, this pragmatic randomised controlled trial was an intervention delivered by front‐line health workers in their usual work. This can often lead to a reduction in the effect seen because of competing time demands and other challenges of delivery (List, Suskind, & Supplee, 2021). The study included a blind‐rated interview measure of child behaviour, representing the gold standard for assessing children's behaviour, as the parent's descriptions of their children's behaviours are rated against standard criteria by the interviewer, so minimising bias. The trial had a high level of retention (81% at 6 years). For this follow‐up study, we were restricted by the original sample size. As a result, we did not set out to hypothesis test as we would have been underpowered to do so. Instead, we prespecified a longitudinal Bayesian model for the analysis, allowing us to optimise statistical efficacy and present the findings in a more intuitive way. In regard to the economic evaluation, the study benefited from a broad costing perspective (i.e. including not only intervention and medical‐related costs but also accommodation, social services, education‐based services and parenting‐related services), direct measurement of health‐related quality of life and a relatively long time horizon to capture impacts of the intervention, compared with previous studies.

Regarding limitations, there was 19% loss to follow‐up. However, alongside the primary analysis that includes a high proportion of those randomised (98%) under a missing at random assumption, we also undertook extensive sensitivity analysis with differing assumptions. These results suggest that we are likely to have underestimated the effect size of the impact of the VIPP‐SD intervention, rather than overstating it. The use of a Bayesian approach, rather than the more commonly used frequentist approach (with *p*‐values), was prespecified and used, in part, because we had a fixed sample size for this follow‐up study, with more limited power. This makes interpretation somewhat more complex, but an 86% probability of a sustained beneficial effect of ViPP‐SD 6 years after intervention nonetheless represents the best available evidence for a brief, early intervention for behavioural problems available. Services will have to decide whether a probability of 86% gives them confidence to offer this intervention compared with other possible approaches, though it is worth noting that this evidence is more substantial than that available for other brief, early life interventions for children at risk of behavioural problems.

The primary effect size of 0.11 could be considered a modest effect for an experimental study, but the literature increasingly suggests that even small effect sizes could have profound effects if they occur in the general population (Carey, Ridler, Ford, & Stringaris, 2023) leading to substantial benefits for child behaviour and longer term health. Thus, researchers examining long‐term effects of population health interventions should expect much smaller (including very small) effect sizes, which can represent actionable evidence given the large benefit when delivered across a population (Matthay et al., 2021).

To our knowledge, this study is the first evidence of sustained benefit of a brief intervention (6 sessions) delivered to parents of young children at risk of developing behaviour problems. Most other comparable studies in young children have been of much longer, more intensive interventions; thus, this finding is particularly noteworthy, as briefer interventions have a larger scope to be delivered at scale. We cannot know with high confidence why VIPP‐SD affects behaviour and whether other brief interventions might be similarly effective. We suspect (and this is conjecture, though other research studies would support) that it has a lot to do with the focus on early parent–child relationships, promoting positive, sensitive parenting, *as well as* focusing on challenging behaviours given the young age of the children (Hutchings, Williams, & Leijten, 2023).

There is now a substantial body of evidence demonstrating that behavioural problems in childhood are associated with a range of longer term adverse outcomes for these children as they grow through adolescence and adulthood (Colman et al., 2009; Offord & Bennett, 1994; Rissanen et al., 2022). Whilst behavioural problems in childhood do not determine a future of problems, these increased risks mean that there is a significant opportunity to prevent problems early in life. The findings of this study add an important chapter to this work, in part because this demonstrates that a brief intervention delivered when children are aged 1–2 years can have sustained benefit at least into middle childhood, with likely benefit extending into the future for these children and their families. Although these findings are encouraging and suggest that short programs like VIPP‐SD delivered in early childhood have an impact, such programs may often need follow‐up with subsequent, age‐relevant interventions. Such follow‐on interventions could be thought of as ‘booster shots’ against behavioural problems and could be particularly relevant for the most vulnerable children and families (see Pingault, Rijsdijk, Zheng, Plomin, & Viding, 2015).

## Conclusion

The findings of this pragmatic RCT and 6‐year follow‐up suggest that there are sustained benefits of the VIPP‐SD intervention on children's behaviour. The magnitude of change is small 6 years postintervention but is consistent with the positive earlier trial findings when the children were aged 2 years and 4 years old. This longer term follow‐up also suggests benefit for parental mental health – something not seen in our earlier follow‐up. These findings suggest that brief, focused early intervention can have cascading benefits through the years for children and their families – something talked about in the field, but not demonstrated much before. This brief intervention offers a greater opportunity to implement at scale. This may also generate economies of scale, for example by embedding training and supervision within routine practice, which would improve the cost‐effectiveness advantage of VIPP‐SD.

## Funding

The National Institute of Health Research Health Technology Assessment programme funded the research (grant number NIHR132896). The funders of the study had no role in the study design, data collection, data analysis, data interpretation or writing of the report.

## Ethical considerations

Ethics approval for the study was given by London‐Surrey NHS Research Ethics Committee (22/LO/0182) on 10 May 2022. The study was registered with the Integrated Research Approval System (IRAS) under the reference number 310487. The sponsor for the study was the University of Cambridge.

**Key points**

What's known?

- Behavioural problems in early childhood are linked to an increased risk of adverse outcomes in adolescence and adulthood. We have previously shown that the brief, six‐session VIPP‐SD intervention can lead to reduced behavioural problems in children, evident up to 24 months later.

What's new?

- In this 6‐year follow‐up study, we showed that there was a probability of 86% that VIPP‐SD led to increased reductions in behavioural problems in children, compared to those whose parents did not receive the intervention. Although the effect size found was small, these findings suggest that brief, focused early intervention can have cascading benefits through the years for children and their families – something talked about in the field, but not demonstrated much before.

What's relevant?

- Brief interventions also offer greater opportunity for implementation at scale, with potential for wider public health benefit and economies of scale, thus improving potential cost‐effectiveness.

## Supporting information


**Appendix S1.** Additional information on study management, measures and sample size calculations.
**Table S1.** Estimated posterior probabilities under the three scenarios submitted in the NIHR HTA grant.
**Table S2.** Estimated intervention effect at 6‐year follow‐up from sensitivity analysis for the primary outcome.
**Appendix S2.** Economic evaluation methods.
**Table S3.** Unit costs and sources.
**Table S4.** Use of health and social care services from baseline to 6‐year follow‐up: 3‐month CA‐SUS.
**Table S5.** Use of health and social care services from baseline to 6‐year follow‐up: 3‐year CA‐SUS.
**Table S6.** Mean costs (£) and outcomes between baseline and 6‐year follow‐up: 3‐month CA‐SUS.
**Figure S1.** Bootstrapped mean differences in costs and QALYs at 6‐year follow‐up: 3‐month CA‐SUS.
**Figure S2.** Cost‐effectiveness acceptability curve showing the probability that VIPP‐SD is cost‐effective compared with usual care at different values of willingness to pay thresholds per QALY gained at 6‐year follow‐up: 3‐month CA‐SUS.
**Figure S3.** Bootstrapped mean differences in costs and PACS at 6‐year follow‐up: 3‐month CA‐SUS.
**Figure S4.** Cost‐effectiveness acceptability curve showing the probability that VIPP‐SD is cost‐effective compared with usual care at different values of willingness to pay thresholds for a 1‐point improvement in PACS score at 6‐year follow‐up: 3‐month CA‐SUS.
**Figure S5.** Bootstrapped mean differences in costs and QALYs at the 6‐year follow‐up: multiple imputation based on 3‐month CA‐SUS.
**Figure S6.** Cost‐effectiveness acceptability curve showing the probability that VIPP‐SD is cost‐effective compared with usual care at different values of willingness to pay thresholds per QALY gained at 6‐year follow‐up: multiple imputation based on 3‐month CA‐SUS.
**Figure S7.** Bootstrapped mean differences in costs and QALYs at the 6‐year follow‐up: 3‐year CA‐SUS.
**Figure S8.** Cost‐effectiveness acceptability curve showing the probability that VIPP‐SD is cost‐effective compared with usual care at different values of willingness to pay thresholds per QALY gained at 6‐year follow‐up: 3‐year CA‐SUS.
**Appendix S3.** Detail on estimands.

## Data Availability

Deidentified participant data and a data dictionary will be made available to bona fide researchers after approval of a proposal, with a signed data access agreement, following a 1‐year period after the publication of this work.
