# Supplementary material for: Long‐term follow‐up of a randomised controlled trial of a brief home‐based parenting intervention to reduce behavioural problems in young children
Source: J Child Psychol Psychiatry. 2025 Sep 17;67(3):321–32. doi: 10.1111/jcpp.70037 (PMC12883591; doi:10.1111/jcpp.70037)
Supplement: Supplementary file 1 — Appendix S1. Additional information on study management, measures and sample size calculations. Table S1. Estimated posterior probabilities under the three scenarios submitted in the NIHR HTA grant. Table S2. Estimated intervention effect at 6‐year follow‐up from sensitivity analysis for the primary outcome. Appendix S2. Economic evaluation methods. Table S3. Unit costs and sources. Table S4. Use of health and social care services from baseline to 6‐year follow‐up: 3‐month CA‐SUS. Table S5. Use of health and social care services from baseline to 6‐year follow‐up: 3‐year CA‐SUS. Table S6. Mean costs (£) and outcomes between baseline and 6‐year follow‐up: 3‐month CA‐SUS. Figure S1. Bootstrapped mean differences in costs and QALYs at 6‐year follow‐up: 3‐month CA‐SUS. Figure S2. Cost‐effectiveness acceptability curve showing the probability that VIPP‐SD is cost‐effective compared with usual care at different values of willingness to pay thresholds per QALY gained at 6‐year follow‐up: 3‐month CA‐SUS. Figure S3. Bootstrapped mean differences in costs and PACS at 6‐year follow‐up: 3‐month CA‐SUS. Figure S4. Cost‐effectiveness acceptability curve showing the probability that VIPP‐SD is cost‐effective compared with usual care at different values of willingness to pay thresholds for a 1‐point improvement in PACS score at 6‐year follow‐up: 3‐month CA‐SUS. Figure S5. Bootstrapped mean differences in costs and QALYs at the 6‐year follow‐up: multiple imputation based on 3‐month CA‐SUS. Figure S6. Cost‐effectiveness acceptability curve showing the probability that VIPP‐SD is cost‐effective compared with usual care at different values of willingness to pay thresholds per QALY gained at 6‐year follow‐up: multiple imputation based on 3‐month CA‐SUS. Figure S7. Bootstrapped mean differences in costs and QALYs at the 6‐year follow‐up: 3‐year CA‐SUS. Figure S8. Cost‐effectiveness acceptability curve showing the probability that VIPP‐SD is cost‐effective compared with usual care at diffe [file JCPP-67-321-s001.docx]

**Appendix S1 – additional information on study management, measures and sample size calculations.**

**Data management**

The study’s source data included paper forms, questionnaires, written interview notes, scoring from the researchers’ research assessments, video recordings, audio recordings, and online forms. Data were entered on an electronic REDCap database. Access to the system was restricted to trained staff with unique password-protected accounts. Identifiable data were not recorded on the REDCap database and participants were identified only by a unique study identifier.

All data monitoring and cleaning were completed on REDCap. The study data was reviewed by the study manager and study statistician for discrepancies and errors. The database was initially soft locked for final data checks to be performed by the study statistician before the hard lock was completed. All outstanding queries from the study statistician were resolved prior to the database hard lock. All statistical analysis was performed using Stata 17.

**Additional secondary outcome measures**

***Patient Health Questionnaire-9***

Self-reported depression severity and symptomatology of caregivers were measured using the Patient Health Questionnaire-9 (PHQ-9). This is a widely used and reliable measure of depression severity. Each of nine statements is scored for the frequency in which the caregiver has experienced each problem over the past two weeks. Scores range from 0 (not at all) to 3 (nearly every day). A higher total score (range 0-27) indicates more severe depressive symptoms.

***Generalised Anxiety Disorder-7***

Caregiver anxiety was assessed using the Generalised Anxiety Disorder-7 (GAD-7). This measure has been used extensively in research as a general measure of anxiety in adults. The GAD-7 is a seven-item questionnaire which asks respondents how often they have experienced each problem in the past two weeks. Each statement is scored from 0 (not at all) to 3 (nearly every day), with a total score (range 0-21) obtained by summing all items of the questionnaire.

***Revised Dyadic Adjustment Scale***

Relationship adjustment was measured using the Revised Dyadic Adjustment Scale (RDAS). The RDAS is a fourteen-item questionnaire. Scores range from 0 to 69, where higher scores indicate greater relationship satisfaction. The RDAS was completed by the caregiver if they were in a relationship.

***Brief Parental Self-Efficacy Scale***

The Brief Parental Self-Efficacy Scale (BPSES) is a 5-item measure of parental self-efficacy. Items are scored on a five-point Likert scale (1 = strongly disagree; 3 = neutral; 5 = strongly disagree), with scores ranging from 5 to 25 with higher scores indicating higher levels of parental self-efficacy.

**Additional information**

**Study organization and management**

***Study management***

The study was overseen by two main oversight committees: (1) Study Steering Committee and (2) Project Management Group. A patient and public involvement (PPI) group was also convened.

***Study Steering Committee***

The Study Steering Committee (SSC) provided overall supervision of the study, monitoring the progress of the study, ensuring there were no major protocol deviations, and provided advice to the study investigators. The SSC comprised an independent chairperson, two PPI members and four additional independent members, as well as non-independent members, including the Co-Chief Investigators, the study manager, and the senior study statistician. Membership of the SSC was approved by the NIHR Health Technology Assessment (HTA) programme prior to convening the committee. The SSC met prior to the commencement of the study and at regular intervals during the study (approximately every 5 months).

***Project Management Group***

The Project Management Group (PMG) was responsible for overseeing the management of the study and operational issues. The PMG met approximately every 2 to 3 months during the study. Members of the PMG included the Co-Chief Investigators, investigators, the study manager, and the study statistician.

**Patient and public involvement group**

Our Patient and Public Involvement (PPI) group was composed of five adult representatives and eight child representatives. Group members were the caregivers and their children aged 6-9 years old. The PPI group contributed to the study’s progress throughout and provided significant and important input on the design and management of the study. The group met online five times during the study, with a minimum of two adult members and three child members attending each meeting.

The PPI group provided ideas, advice and feedback that were highly valuable to the study’s recruitment process, participant retention, data collection procedures, and dissemination of the study findings. The group advised and offered feedback on communication with families, including suggestions for reaching out to families that the research team had struggled to re-engage with recruitment for the follow-up study. The PPI group advised on key engagement strategies, such as a thank you toy for the study children and development of regular study newsletters for participant families (including pictures of the team so children can familiarise with before their visit). The group were also involved in decisions around how to communicate the study findings to participants.

The PPI members were engaged and motivated throughout the study. At the start of each meeting, a summary of the study progress was provided. The child members were kept engaged in the meetings by using age-appropriate language, asking them specifically for their feedback on aspects of the study (e.g., selection of the thank you gift for the study children), and including playful elements in the meeting (e.g., a game at the end). Two members of the PPI group were also members of the PMG. All PPI members were sent a voucher for each meeting they attended in line with the INVOLVE guidelines.

**Amendments to protocol**

There were no amendments made to the study protocol following approval of the protocol by the ethics committee.

**Sample Size**

A total sample of 300 have been recruited to the trial from 7 sites across London and the East and Central England. There are 296/300 (98%) children with outcome data collected at one or both post-intervention time points (5 and 24 months). These 296 participants have been included in the primary analysis of this follow-up trial by means of a longitudinal model. We achieved a very high retention rate at 24 months of n=282/300 (94%), and we anticipate a minimum 85% retained sample at 6-year follow-up (n≈256).

The sample size is fixed. In order to obtain most value from the trial we used a Bayesian analysis framework as this permits a different context for interpretation around the strength of the evidence. A Bayesian analysis enabled us to calculate the posterior probability for superiority of our intervention. Posterior probabilities are not only more intuitive to interpret compared to p-values but this framework discourages the study from being inappropriately evaluated in a rigid frequentist hypothesis-testing framework, which can be unsuitable when the sample size is limited. When there is no control over the sample size there can be a temptation is to assume an overly optimistic minimum clinically important effect size, an academic exercise tailoring the power calculation to fit.  When the sample size is restricted and analysed in a frequentist framework the study is vulnerable to misinterpretation. There would be great value in the result of this trial even if the p-value is ‘borderline’. Borderline p-values are difficult to interpret and are frequently misinterpreted. In this application we demonstrate what strength of evidence can be gained from this trial through simulation. We used a fixed sample size of n=296 in the analysis model (with n=282 at 24 months and n ≈256 at 6 years) so that funding committee members can judge the value of undertaking this study by examining the potential strength of randomised evidence the study can provide.

We examined three scenarios for the potential long-term intervention effect size. **Note that we assume an effect size of 0.22 as this is the intervention effect estimated using a longitudinal model and is the model we used in this follow-up analysis as opposed to the HSHS original model that was single time point only.**

1. Where the intervention effect size is **sustained** at 6 years i.e. it is similar to the 24-month result which found a reduction of 2.10 on the PPACS (ES 0.22) – this is the most likely outcome based on existing published evidence.
2. Where the intervention effect size has **diminished** by 50% (ES 0.11)
3. Where the intervention effect size **disappeared** (ES of 0.0)

**Sample size simulation**

As we already have the data for baseline, 5 month and 24-month outcome from children, the data-generating model was constructed using a mixed effects linear regression model with subject specific effects (random slope and random intercept). The model included two time points, group, group by time interaction, baseline value and center. Outcome data at 6 years follow-up were

simulated for each scenario 250 times based on the mixed effects model with a random draw from a Gaussian distribution using a standard deviation calculated from of the variance covariance matrix of the longitudinal model. In each simulation the control arm participants had 5 year predicted outcome calculated based on their 24-month observed value and random draws from N(0, 6.38). The same approach was used for those in the active arm, but with the distribution for the random draws having different mean values for the three scenarios; sustained effect N(0, 6.38 ); diminished effect N(1.05,  6.38)**;** effect disappeared (N(2.10, 6.38).

Posterior probabilities were estimated for each simulation using a Bayesian longitudinal model with 20,000 MCMC samples, thinning every 4 to provide 5000 MCMC samples. Covariates for time, group, group by time interaction and baseline score were included with uninformative Gaussian prior distributions on all model parameters (N(0, 1000)). The model included 294 participants with post-baseline data but outcome values were randomly deleted at the 6-year timepoint so that, on average, only 85% (n=254) of participants contributed to the model at this timepoint to reflect the expected proportion of missing data in the follow-up.

**Simulation results**

Table S2 (see below) displayed the average posterior probability under each scenario for superiority and also a conservative approach for superiority with a margin, with a margin of an intervention effect being at least a mean difference of 1 on the PACS score.

**If the intervention effect is sustained simulations demonstrate that with the sample size available in this trial we would find an average probability of superiority of 0.931 i.e. 93%.** If the effect size is reduce by half (diminished) the probability of superiority will on average be 0.779, (78%). If it has disappeared then we expect to see reassuringly low probabilities of around 0.48 (48%).To enhance inference we have also examined the probability of the intervention effect (mean difference in PACS) being at least greater than one.

**Table S1. Estimated posterior probabilities under the three scenarios submitted in the NIHR HTA grant.**

|  | Average **probability** **of superiority** of ViPP  (Mean PACs between arm difference  > 0) | Average **probability** **of superiority with a margin** of ViPP (Mean PACs between arm difference  > 1 ) |
| --- | --- | --- |
| **Sustained** effect (ES of 0.22) | 0.931 | 0.767 |
| **Diminished** effect (ES of 0.11) | 0.779 | 0.512 |
| **Disappeared** effect (ES of 0.00) | 0.480 | 0.215 |

**Sensitivity analysis for primary outcome**

**Table S2. Estimated intervention effect at 6-year follow-up from sensitivity analysis for the primary outcome**

| **Sensitivity** | **Intervention effect estimate** | **95% Credible Interval** | **Posterior Probability of Superiority** |
| --- | --- | --- | --- |
|  |  |  |  |
|  |  |  |  |
| **All Participants Included** | **-0.19** | **(-0.41 to 0.03)** | **0.95** |
| All Missing Worse | -0.17 | (-0.39 to 0.04) | 0.94 |
| VIPP-SD Worse | -0.15 | (-0.36 to 0.07) | 0.91 |
| Control Better | -0.16 | (-0.38 to 0.05) | 0.93 |
| Control Worse | -0.21 | (-0.43 to 0.00) | 0.97 |

**Appendix S2. Economic evaluation methods**

**Service Use**

Given the long length of follow-up since the end of the original RCT, and concerns about the ability of caregivers to recall all service use in detail over this time period, two versions of the Child and Adolescent Service Use Schedule (CA-SUS) were developed.

First, a comprehensive CA-SUS was developed, which covered the full range of services collected in the original RCT (all health and social care services relevant to the population) but only asked caregivers to report over a 3-month period to aid recall (herein known as the ‘3-month CA-SUS’). Services covered in the 3-month CA-SUS included accommodation services (including residential care and formal and informal foster care and kinship), hospital inpatient and outpatient services (including those related to accidents, mental health and physical health conditions), A&E and ambulance services, a wide range of community-based health and social care services (including contacts with GPs, nurses, mental health workers, psychological therapists, speech & language therapists, portage services, special education needs co-ordinators, art, drama, music or occupational therapists, social workers, youth workers and family support workers, and parent training/parenting courses), and prescribed medication for mental health problems (e.g. for depression, anxiety, ADHD, etc.).

The second CA-SUS was a less comprehensive, briefer version focused only on key services (those that are high cost and/or high use) which are more easily recalled, but it asked caregivers to report the use of these key services over the full period between the 2-year and the 6-year follow-up points (herein known as the ‘3-year CA-SUS’). Please note that the exact length of follow-up was not known in advance and was dependent on start date of the interviews as well as the speed with which participants agreed to be followed up and interviews were undertaken. The team initially expected follow-ups to be approximately 5 years post-randomisation (hence the ‘3-year CA-SUS’ terminology), but in the end the average length of follow-up was closer to six years post-randomisation. We have retained the original terminology used to refer to the CA-SUS but stress that respondents were asked to report service use ‘since you were last interviewed’, rather than over a specific period of time.

The 3-year CA-SUS covered a narrower range of services, including accommodation (foster, kinship and residential care), hospital inpatient, outpatient, A&E and ambulance services, and a small number of community health services (GPs, practice nurses, mental health workers, psychological therapists, and parent training/parenting courses).

The 3-month CA-SUS was thus comprehensive but did not cover the full period from the 2-year to the 6-year interview and focused instead on recent service use. However, the short recall period is likely to ensure greater accuracy than the 3-year CA-SUS. For this reason, the 3-month CA-SUS was used for the base case (the primary) economic analysis, with the 3-year CA-SUS tested in sensitivity analysis. Services in the 3-month CA-SUS were costed and multiplied up to provide a cost for the full period from 2-year to 6-year follow-up, which assumes that use of services over time is linear and that differences in the use of services between the two groups in the 3-months prior to the 6-year interview, reflects differences over the full period from 2-years to 6-years.

**Estimation of costs**

In the original HSHS RCT, unit costs were for the financial year 2017/18. Within the HSHS RCT, the cost of the VIPP-SD intervention was directly calculated using a standard micro-costing approach in the HSHS RCT (for detailed information, see (O’Farrelly, Barker, et al., 2021) and included therapist salaries (including employer on-costs and appropriate overheads), therapist training and supervision and equipment. All other unit costs were identified from nationally relevant sources, as outlined in the HSHS RCT report (O’Farrelly, Barker, et al., 2021). For consistency, we inflated these unit costs to 2021/22 prices using the NHS cost inflation index (Jones et al., 2023) and applied them to the service use reported in the follow-up study (summarised in Table S2). We combined the follow-up costs (by sector and in total) with costs from the HSHS RCT (also inflated to 2021/22 prices) to calculate costs covering the full follow-up period from baseline to 6-year follow-up. For the 3-month CA-SUS, this included the original RCT costs plus the 3-month costs multiplied up to cover the full 2-year to 6-year follow-up period. For the 3-year CA-SUS, this included only the narrower range of key services recorded at follow-up, and thus required the same narrower range of services to be extracted from the original RCT. All costs are reported in pounds sterling for the financial year 2021/22. In line with NICE recommendations (NICE 2023), costs accruing beyond year one were discounted using an annual discount rate of 3.5%.

**Outcomes**

QALYs were calculated using data from the CHU9D measure of health-related quality of life (Stevens, 2009) estimated directly at the 6-year follow-up or estimated via mapping from the SDQ (Furber, Segal, Leach, & Cocks, 2014) for the HSHS RCT timepoints. CHU9D health states were converted to utility scores by applying quality weights (Stevens, 2012) and utility for each year of follow-up was estimated assuming a linear change in utility score over time. QALYs were then calculated using the area under the curve approach (Manca, Hawkins, & Sculpher, 2005) and discounting at 3.5% per annum was applied to QALYs accrued after year one, as recommended by NICE (2023)

**Data analysis**

The base case economic analysis was a complete case analysis, with effects measured in terms of QALYs and costs estimated from the 3-month CA-SUS. We conducted two sensitivity analyses to test the robustness of these base-case results. Firstly, multiple imputation with chained equations and predictive mean matching were used to assess the impact of missing data. This analysis was based on 28 multiply imputed data sets of total costs and outcomes. Secondly, we assessed cost-effectiveness in terms of QALYs using costs based on the 3-year CA-SUS.

The datasets were combined, managed and analysed using STATA 18 (StataCorp, 2023). Health and social care service use was described by trial group as the mean, standard deviation (SD), range and the percentage of participants who reported at least one contact over the 6-year follow-up. Differences between service use were not tested statistically to avoid issues with multiple significance testing and because the focus of the economic evaluation was on the costs and cost-effectiveness.

For information, and to support understanding of the cost and outcome data analysed in the economic evaluations, costs and outcomes are initially analysed separately using standard frequentist approaches (linear regression models, as outlined in the main paper). However, standard approaches to cost-effectiveness/cost-utility analyses are Bayesian in nature, exploring the probability that one intervention is cost-effective compared to another, given the data available (Briggs, 1999).

**Economic results**

***Service use***

Service use over the follow-up period from baseline to 6-year follow-up is reported in Table S 3 for the 3-month CA-SUS and Table S4 for the 3-year CA-SUS. Services used most frequently included contacts with GPs and nurses, A&E attendances and outpatient appointments. Inpatient nights, ambulance attendance, parent training courses and contacts with speech and language therapists and special education needs co-ordinators/educational psychologists were also used relatively frequently (by more than 10% of both groups). The percentage of participants using each service and the mean use of each service was similar between the two groups, with no clear differences or consistent pattern of greater use in one group compared to another.

***Costs***

Mean costs per participant over the follow-up period from baseline to 6-year follow-up are presented in Table S5 for the 3-month. Hospital, community health and social care services and accommodation services were all lower cost in the VIPP-SD group compared to usual care, but total costs were higher for VIPP-SD when the cost of the intervention was included (adjusted mean difference £1,479, 95% CI -380 to 3,338, p=0.0119).

***Outcomes***

QALYs and PACS score for patients with full economic data included in the base case economic analysis are reported in Table S5. QALYs were significantly higher (better health-related quality of life) in the VIPP-SD group compared with usual care (adjusted mean difference 0.044, 95% CI 0.001 to 0.087, p=0.046). PACS scores were also better in the VIPP-SD group although not significantly so (adjusted mean difference -2.54, 95% CI -5.28 to 0.21, p=0.070).

**Table S3. Unit costs and sources**

| Service | Unit cost or range (£) | Source(s) | Notes |
| --- | --- | --- | --- |
| Hospital inpatient (per night) | 529.96-703.63 | NHS Reference Costs, 2017–18 (Department of Health and Social Care, 2018) | Inflated from 2017/18; weighted average of short and long stay |
| Hospital outpatient (per appointment) | 239.57 | NHS Reference Costs, 2017–18 (Department of Health and Social Care, 2018) | Inflated from 2017/18; average outpatient appointment |
| Hospital A&E (per attendance) | 162.15-270.50 | NHS Reference Costs, 2017–18 (Department of Health and Social Care, 2018) | Inflated from 2017/18; varied dependent on use of ambulance services |
| Community-based health and social care (per contact) | 7.36-216.62 | Curtis, 2017 | Inflated from 2017/18; varied by specialist seen |
| Accommodation (per day) | 19.80-100.86 | Curtis, 2017; Gov.uk: foster carers; DirectGov: local housing allowance rates | Inflated from 2017/18; varied by type of accommodation |
| Medication (per week) | 1.96 | Prescription Cost Analysis, 2022 | Assuming £8.50 per prescription per month |

**Table S4. Use of health and social care services from baseline to 6-year follow-up: 3-month CA-SUS**

|  | VIPP-SD  (n=95) | | | Usual care  (n=104) | | |
| --- | --- | --- | --- | --- | --- | --- |
| **Service** | **Mean (SD)** | **Range** | **Percentage**  **using** | **Mean (SD** | **Range** | **Percentage**  **using** |
| **Hospital** |  |  |  |  |  |  |
| Inpatient nights | 0.66 (4.01) | 0 to 37 | 11.70 | 0.30 (1.39) | 0 to 12 | 9.62 |
| Outpatient appointments | 3.69 (8.16) | 0 to 37 | 38.46 | 5.06 (13.64) | 0 to 84 | 40.40 |
| A&E attendances | 2.42 (6.20) | 0 to 52 | 54.74 | 2.30 (4.48) | 0 to 25 | 55.34 |
| Ambulance attendances | 0.37 (1.83) | 0 to 13 | 10.53 | 0.11 (0.34) | 0 to 2 | 9.62 |
| **Community health and social care contacts** |  |  |  |  |  |  |
| GP (at home, surgery or by phone) | 11.07 (13.91) | 0 to 84 | 91.58 | 10.28 (13.09) | 0 to 75 | 85.58 |
| Any nurse | 6.64 (8.67) | 0 to 52 | 95.74 | 6.34 (7.68) | 0 to 37 | 87.50 |
| Parent training course | 3.40 (13.93) | 0 to 96 | 16.84 | 3.86 (14.94) | 0 to 120 | 18.72 |
| Speech and language therapist | 1.80 (8.41) | 0 to 72 | 11.58 | 3.65 (18.14) | 0 to 130 | 20.39 |
| SENCo or educational psychologist | 3.22 (15.89) | 0 to 144 | 10.53 | 8.82 (37.30) | 0 to 288 | 14.42 |
| Any therapist providing ‘talking therapy’ | 5.14 (23.87) | 0 to 156 | 8.60 | 2.01 (10.78) | 0 to 98 | 8.74 |
| Any mental health worker | 1.70 (8.64) | 0 to 72 | 7.45 | 2.64 (15.02) | 0 to 144 | 7.77 |
| Art, drama, music or occupational therapist | 0.59 (2.78) | 0 to 20 | 7.37 | 1.90 (10.36) | 0 to 81 | 5.77 |
| Family support worker | 1.35 (7.94) | 0 to 72 | 6.32 | 2.07 (14.35) | 0 to 144 | 9.62 |
| Social worker or social services youth worker | 0.19 (0.94) | 0 to 6 | 4.21 | 0.95 (4.42) | 0 to 36 | 8.74 |
| **Accommodation nights^a^** |  |  |  |  |  |  |
| Other (e.g. supported housing) | 0.00 (0.00) | 0 to 0 | 0.00 | 1.34 (13.63) | 0 to 139 | 0.96 |

SENCO=Special Education Needs Co-ordinator; a There was no reported use of other accommodation (residential care, foster care, kinship care)

**Table S5.Use of health and social care services from baseline to 6-year follow-up: 3-year CA-SUS**

|  | VIPP-SD  (n=95) | | | Usual care  (n=104) | | |
| --- | --- | --- | --- | --- | --- | --- |
| **Service** | **Mean (SD)** | **Range** | **Percentage**  **using** | **Mean (SD** | **Range** | **Percentage**  **using** |
| **Hospital** |  |  |  |  |  |  |
| Inpatient nights | 0.44 (1.78) | 0 to 16 | 16.84 | 0.66 (1.83) | 0 to 12 | 21.15 |
| Outpatient appointments | 2.31 (3.50) | 0 to 16 | 54.74 | 3.24 (5.98) | 0 to 42 | 62.50 |
| A&E attendances | 2.01 (4.13) | 0 to 38 | 74.74 | 1.94 (2.28) | 0 to 15 | 74.04 |
| Ambulance attendances | 0.22 (0.66) | 0 to 5 | 15.79 | 0.27 (0.67) | 0 to 4 | 18.27 |
| **Community health and social care contacts** |  |  |  |  |  |  |
| GP (at home, surgery or by phone) | 8.86 (9.93) | 0 to 80 | 94.74 | 7.83 (7.67) | 0 to 52 | 93.27 |
| Practice nurse | 3.58 (3.32) | 0 to 21 | 92.55 | 3.46 (2.81) | 0 to 14 | 87.38 |
| Parent training course | 1.20 (3.64) | 0 to 20 | 17.78 | 1.43 (4.71) | 0 to 31 | 21.00 |
| Any therapist providing ‘talking therapy’ | 2.37 (12.72) | 0 to 120 | 15.79 | 1.37 (5.02) | 0 to 31 | 12.62 |
| Any mental health worker | 0.48 (2.52) | 0 to 20 | 9.47 | 1.66 (7.95) | 0 to 72 | 10.58 |

Note: there was no reported use of included accommodations (residential care, foster care, kinship care, etc.)

**Table S6. Mean costs (£) and outcomes between baseline and 6-year follow-up: 3-month CA-SUS**

| Cost category | VIPP-SD  Mean (SD) | Usual care  Mean (SD) | Unadjusted difference^a,b^ | Adjusted difference^a,b,c^ | 95% CI | p-value |
| --- | --- | --- | --- | --- | --- | --- |
| **Costs** |  |  |  |  |  |  |
| Intervention | 1715 (542) | n/a | 1715 | 1708 | 1604 to 1812 | 0.000 |
| Hospital services | 1625 (3551) | 1830 (3594) | -205 | -166 | -1139 to 807 | 0.738 |
| Community-based services | 2680 (5479) | 2773 (5268) | -93 | -318 | -1630 to 994 | 0.635 |
| Medication | 55 (106) | 30 (62) | 25 | 24 | 1 to 48 | 0.045 |
| Accommodation services | 0 (0) | 57 (582) | -57 | -8 | -43 to 28 | 0.673 |
| Total cost | 6074 (7017) | 4689 (7527) | 1385 | 1479 | -380 to 3,338 | 0.119 |
| **Outcomes** |  |  |  |  |  |  |
| QALYs | 4.037 (0.158) | 3.981 (0.201) | 0.056 | 0.044 | 0.001 to 0.087 | 0.046 |
| PACS score | 23.91 (8.81) | 26.52 (11.33) | -2.61 | -2.54 | -5.28 to 0.21 | 0.070 |

a Comparison based on complete case data; b Unadjusted and adjusted differences based on 5,000 bootstrapped replications; c Adjusted for recruitment centre, age of child at follow-up, number of caregivers participating and baseline values (e.g., baseline CHU9D, PACS score, or total cost/total cost for the relevant category)

**Figure S1. Bootstrapped mean differences in costs and QALYs at 6-year follow-up: 3-month CA-SUS**

**
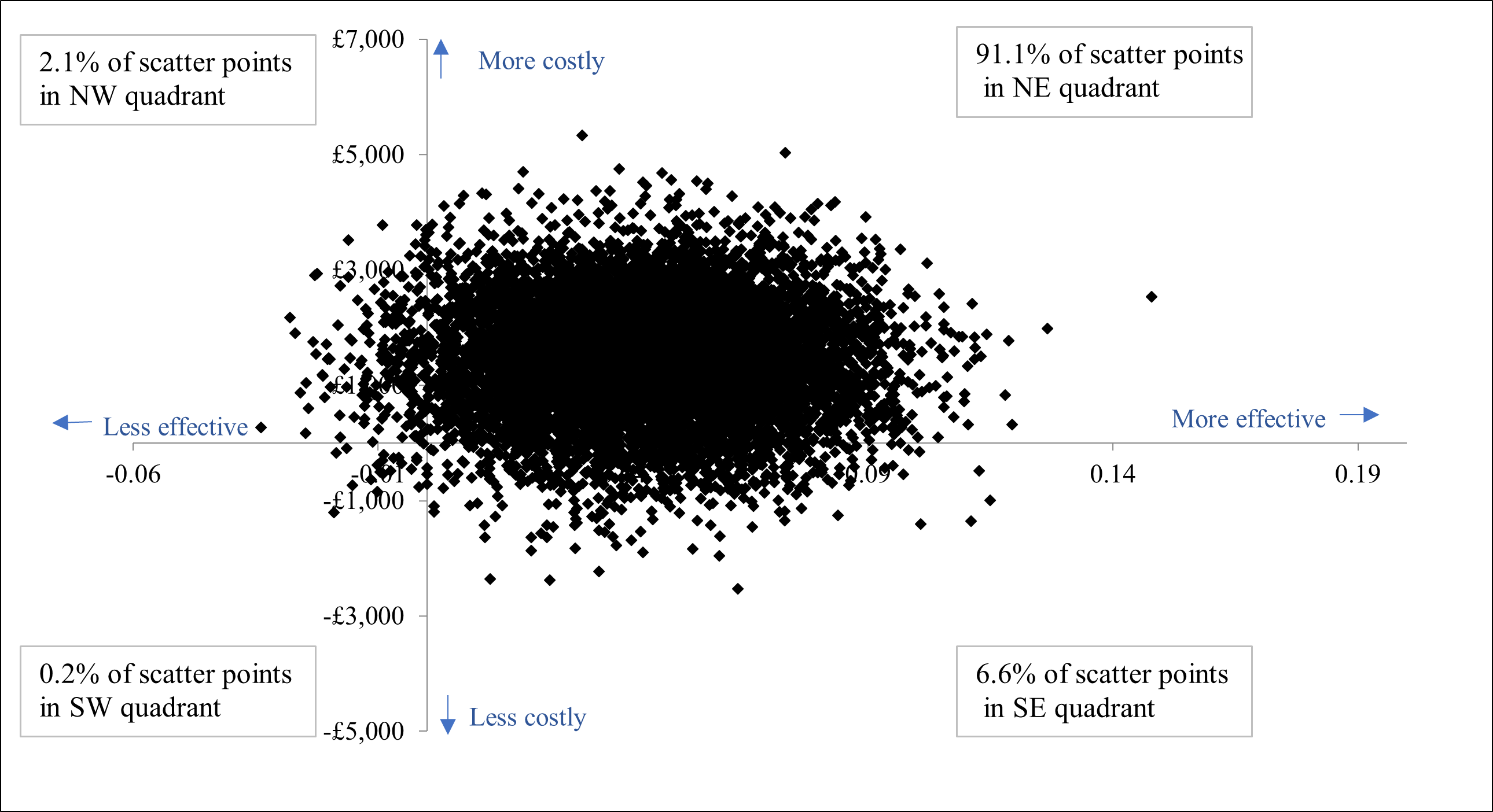
**

Based on 10,000 bootstrapped replications of adjusted model. NE, north-east (more costly, more effective); NW, north-west (more costly, less effective); SE, south-east (less costly, more effective); SW, south-west (less costly, less effective)

**Figure S2. Cost-effectiveness acceptability curve showing the probability that VIPP-SD is cost-effective compared with usual care at different values of willingness to pay thresholds per QALY gained at 6-year follow-up: 3-month CA-SUS**

**
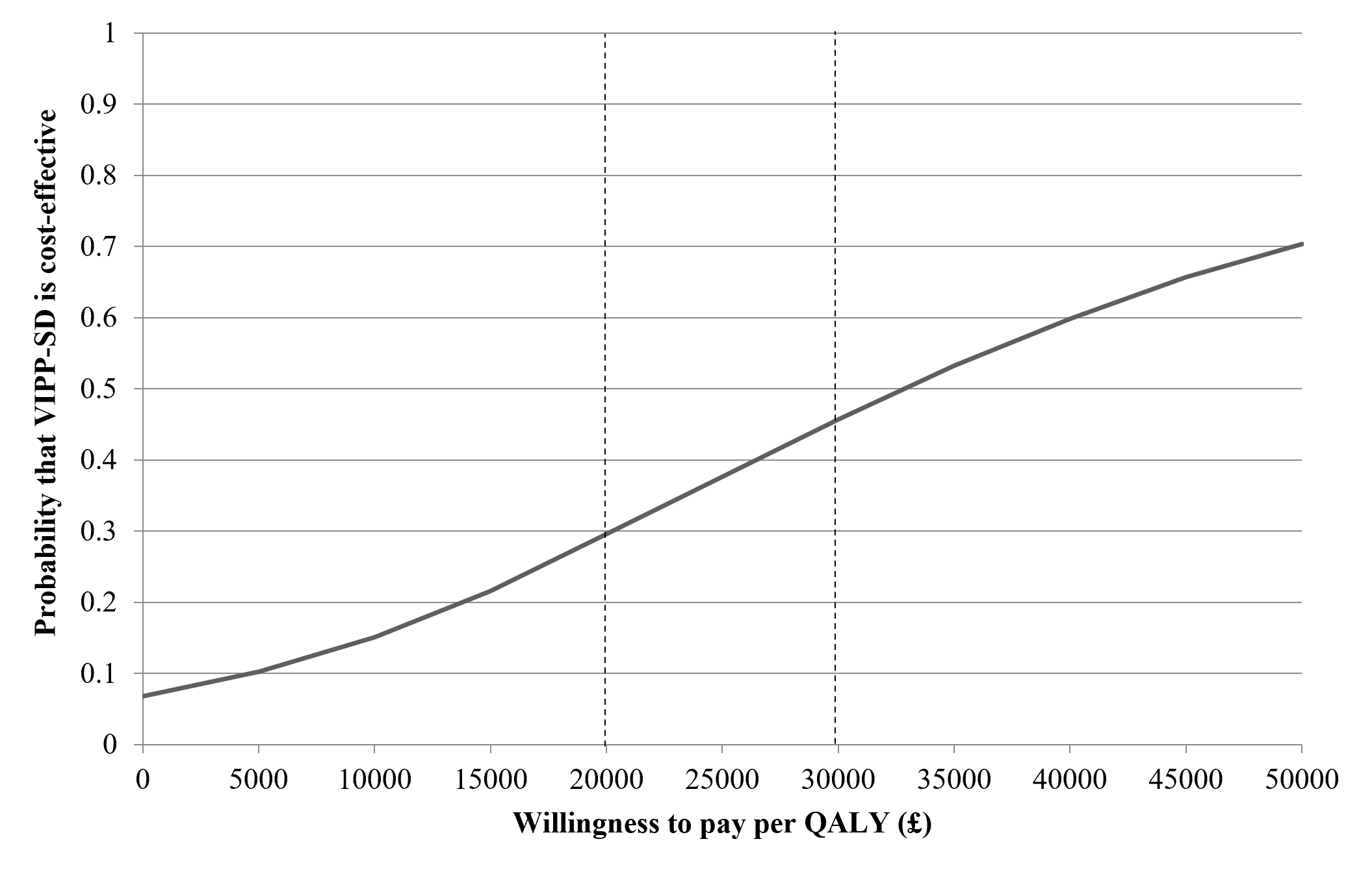
**

**Figure S3. Bootstrapped mean differences in costs and PACS at 6-year follow-up: 3-month CA-SUS**

**
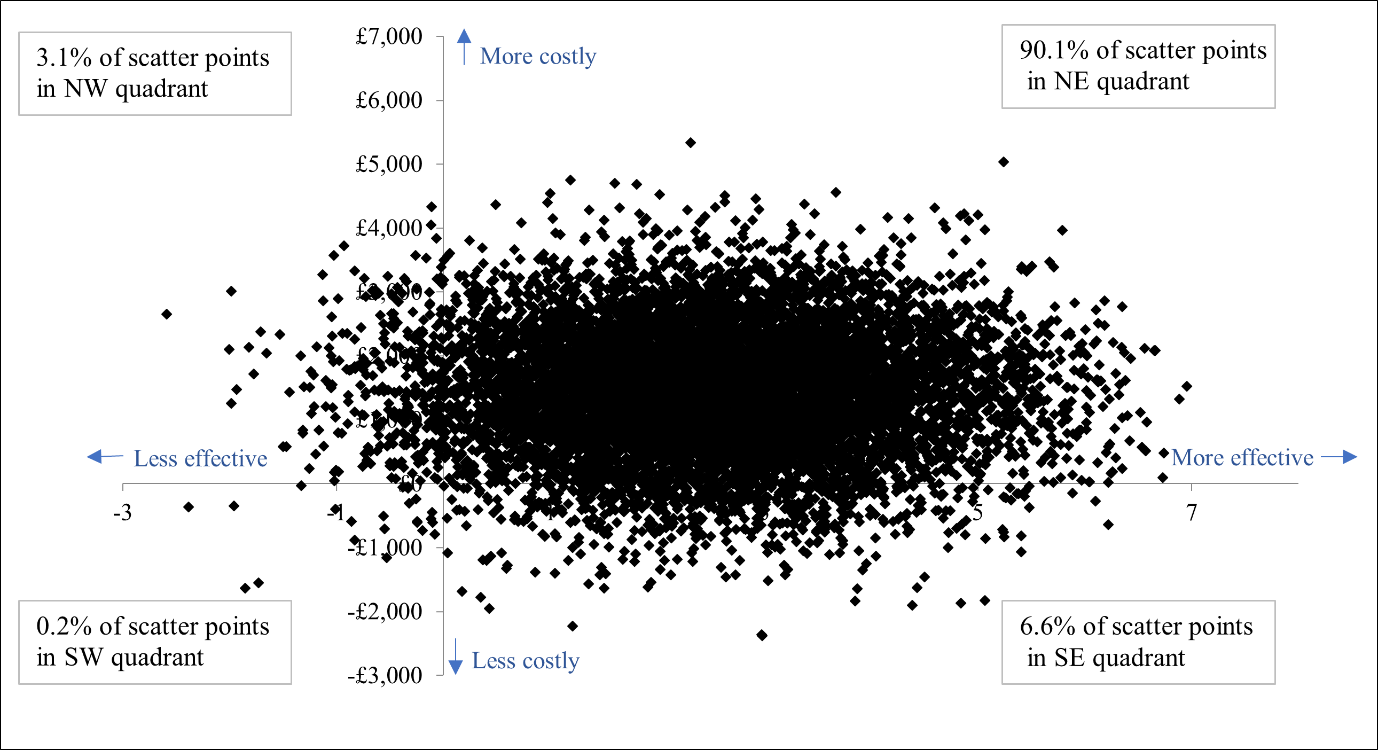
**

**Figure S4. Cost-effectiveness acceptability curve showing the probability that VIPP-SD is cost-effective compared with usual care at different values of willingness to pay thresholds for a 1-point improvement in PACS score at 6-year follow-up: 3-month CA-SUS**

**
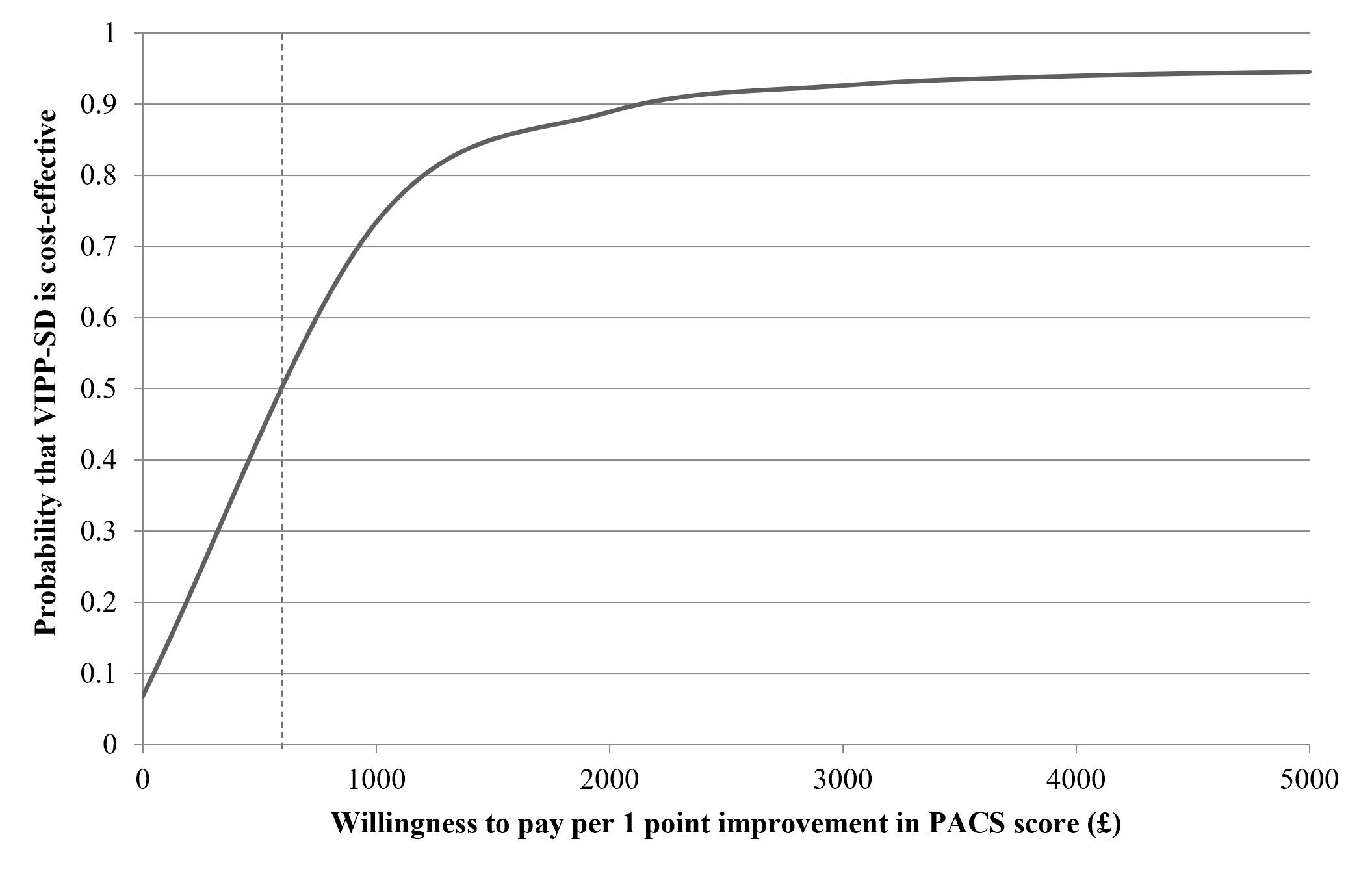
**

**Figure S5. Bootstrapped mean differences in costs and QALYs at the 6-year follow-up: multiple imputation based on 3-month CA-SUS**

**
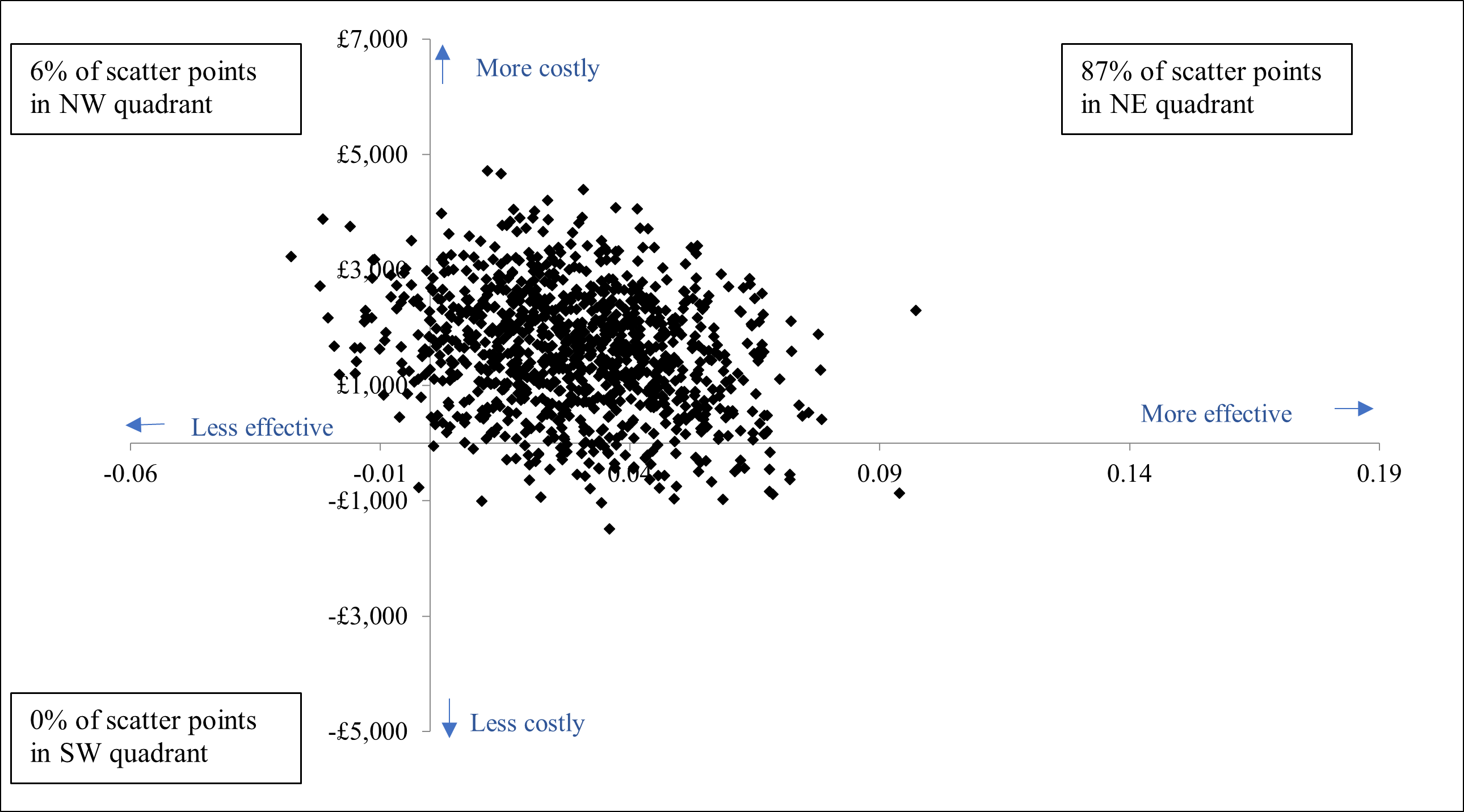
**

**Figure S6. Cost-effectiveness acceptability curve showing the probability that VIPP-SD is cost-effective compared with usual care at different values of willingness to pay thresholds per QALY gained at 6-year follow-up: multiple imputation based on 3-month CA-SUS**

**
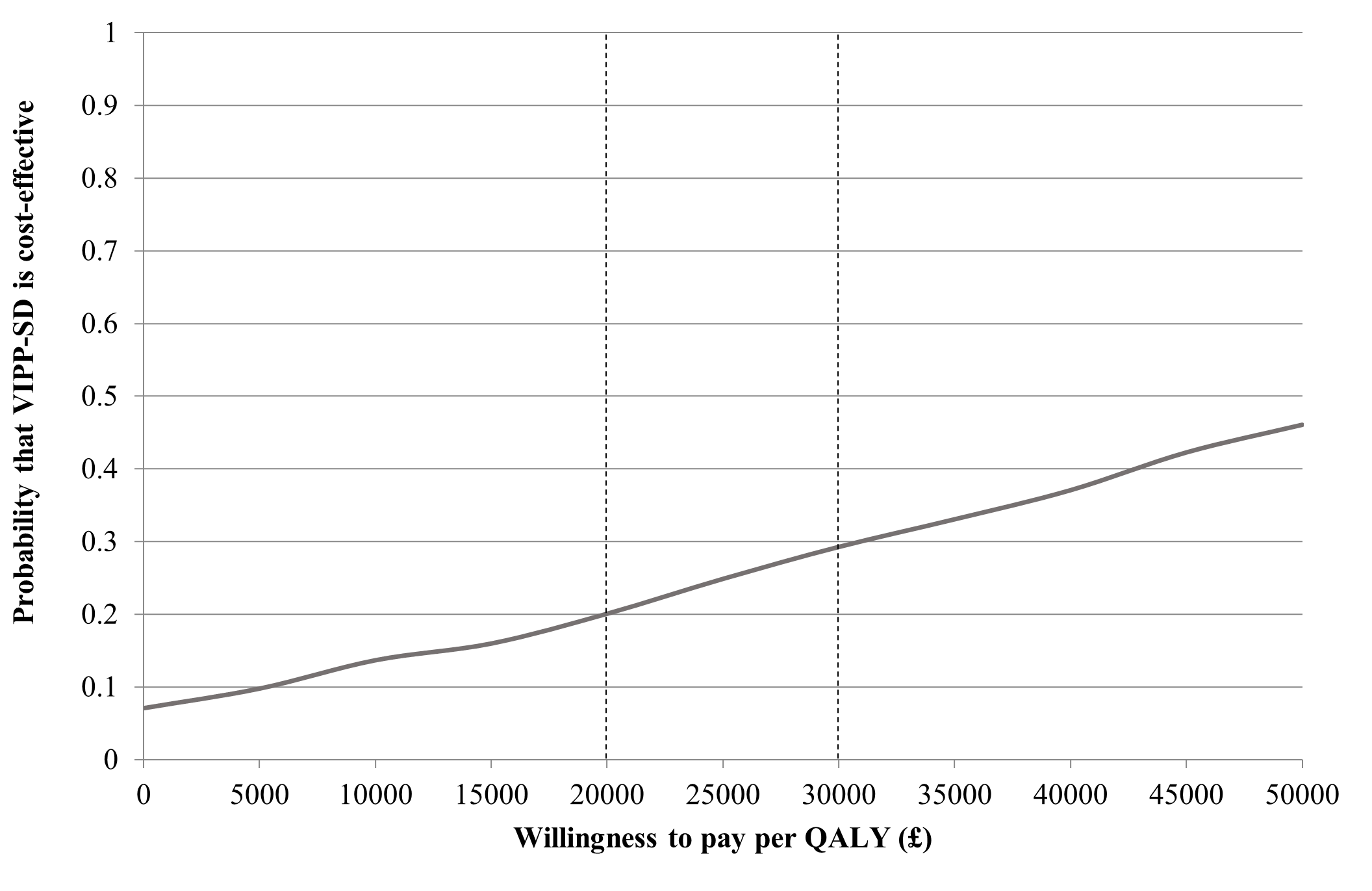
**

**Figure S7. Bootstrapped mean differences in costs and QALYs at the 6-year follow-up: 3-year CASUS**

**
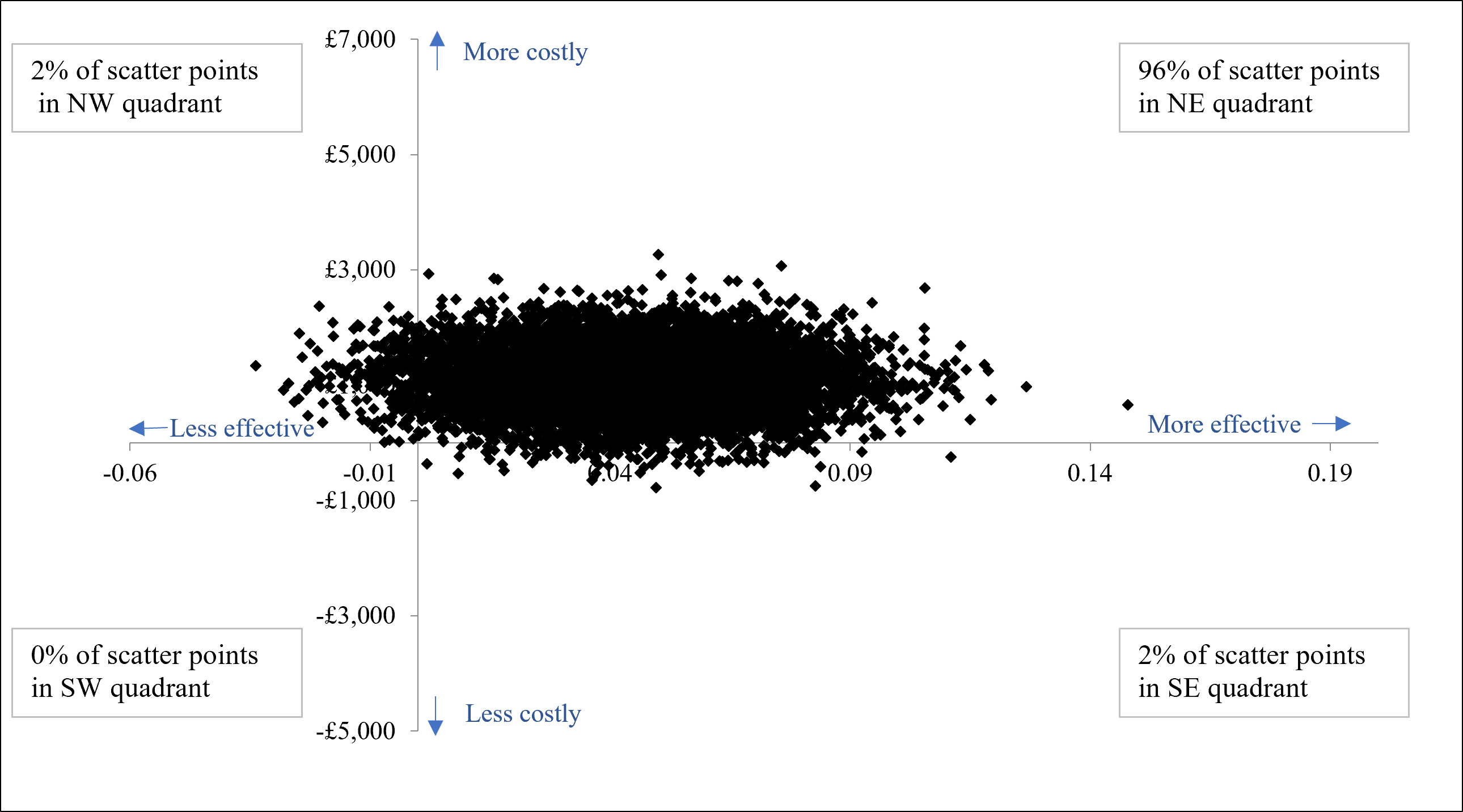
**

Based on 10,000 bootstrapped replications of adjusted model. NE, north-east (more costly, more effective); NW, north-west (more costly, less effective); SE, south-east (less costly, more effective); SW, south-west (less costly, less effective)

**Figure S8. Cost-effectiveness acceptability curve showing the probability that VIPP-SD is cost-effective compared with usual care at different values of willingness to pay thresholds per QALY gained at 6-year follow-up: 3-year CA-SUS**

**
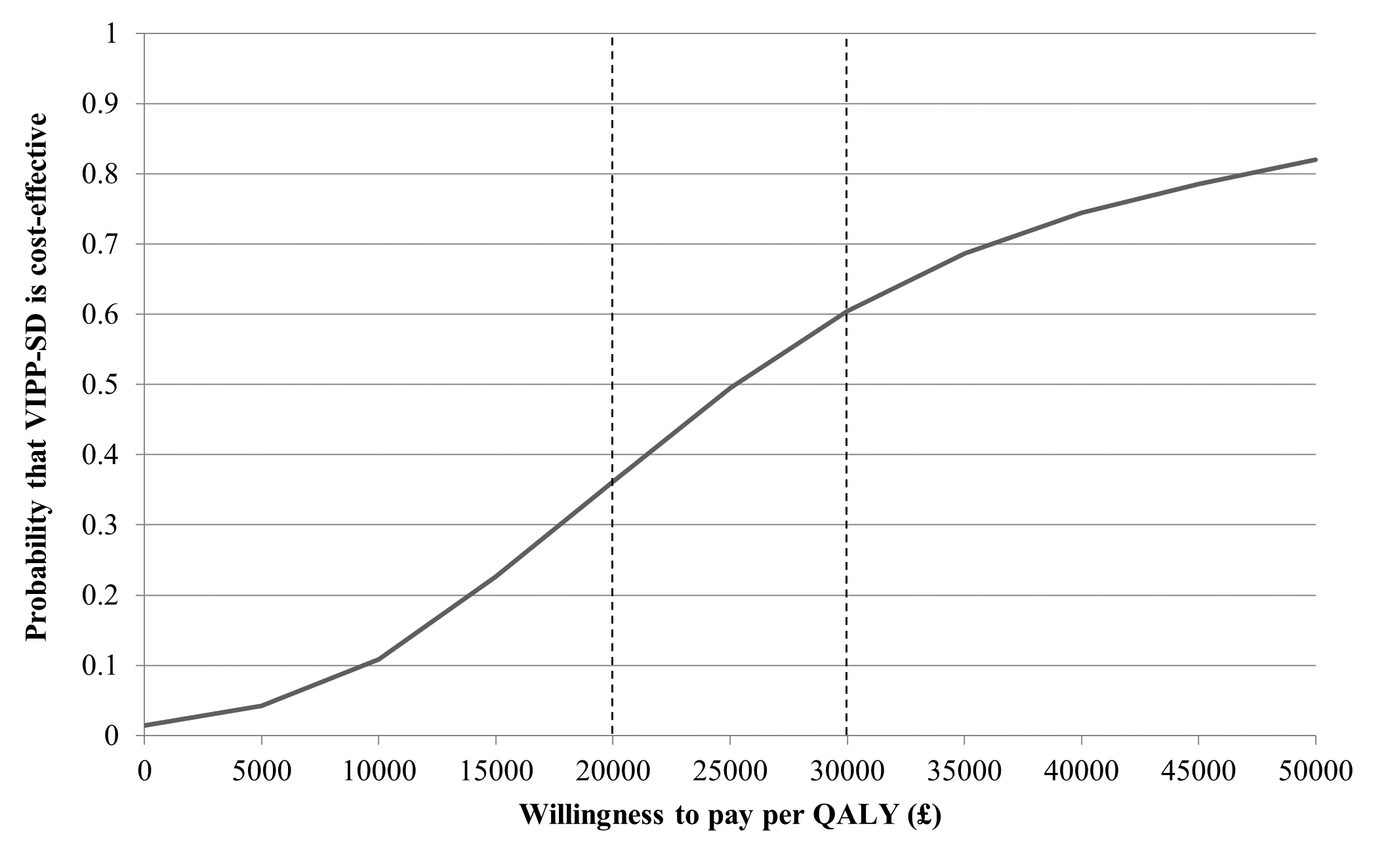
**

**Appendix S3. Detail on estimands.**

***PRIMARY ESTIMAND: Treatment Policy estimand attributes***

| Estimand attribute | Description |
| --- | --- |
| Population | Young children who were aged 12-36 months when recruited to the original HSHS RCT and who had high levels of behaviour problems (originally recruited on the basis of scoring in the top 20% of population norms for the Strengths and Difficulties Questionnaire externalising subscale) and their caregiver(s). |
| Intervention condition(s) | Any use of the VIPP-SD intervention. |
| Variable (outcome) | Standardised total score of Parental Account of Symptoms (PACS) at 5 years post randomisation. |
| Strategies used to handle Intercurrent events | Intervention discontinuation – Treatment Policy  Alternative impactful intervention/s – Treatment Policy  Confirmed Neuro-developmental Difficulties – Treatment Policy |
| Population-level summary measure | Posterior probability of superiority of VIPP-SD compared to usual care adjusted for baseline PACS score and stratification variables (treatment centre & no. parents in study) and child’s age.  This will be reported with the mean difference in standardised PACS score at 5 years post randomisation, between VIPP-SD and usual care |

The estimand will answer the question “What is the probability that ViPP-SD is superior to usual care for behavioural problems in young children, regardless of adherence, use of alternative interventions and Confirmed Neuro-developmental Difficulties , 5 years post randomisation?”

**SUPPLEMENTARY ESTIMANDS ON THE PRIMARY OUTCOME**

| ***Intervention adherence estimand attributes***  Estimand attribute | Description |
| --- | --- |
| Population | Young children who were aged 12-36 months when recruited to the original HSHS RCT and who had high levels of behaviour problems (originally recruited on the basis of scoring in the top 20% of population norms for the Strengths and Difficulties Questionnaire externalising subscale) and their caregiver(s). |
| Intervention condition(s) | Use of at least 4 of the 6 sessions of the Video-feedback Intervention to promote Positive Parenting and Sensitive Discipline (VIPP-SD) intervention. |
| Variable (outcome) | Standardised total score of Parental Account of Symptoms (PACS) at 5 years post randomisation. |
| Strategies used to handle Intercurrent events | Intervention discontinuation – Principal Stratum  Alternative impactful intervention/s – Treatment Policy  Confirmed Neuro-developmental Difficulties – Treatment Policy |
| Population-level summary measure | Mean difference in standardised PACS score at 5 years post randomisation, between VIPP-SD and usual care adjusting for baseline PACS score and stratification variables (treatment centre & no. parents in study). |

The estimand above will answer the question “Is ViPP-SD superior to usual care for behavioural problems in young children, in those that had good adherence and regardless of access alternative interventions or Confirmed Neuro-developmental Difficulties , 5 years post randomisation?”

| ***Alternative Intervention/s estimand attributes***  Estimand attribute | Description |
| --- | --- |
| Population | Participants in the original HSHS RCT |
| Intervention condition(s) | Use of at least 4 of the 6 sessions of VIPP-SD. |
| Variable (outcome) | Standardised total score of Parental Account of Symptoms (PACS) at 5 years post randomisation. |
| Strategies used to handle Intercurrent events | Intervention discontinuation – Treatment Policy  Alternative intervention/s – Principal Stratum  Confirmed Neuro-developmental Difficulties – Treatment Policy |
| Population-level summary measure | Mean difference in PACS score at 5 years since randomisation, between VIPP-SD and usual care  adjusting for baseline PACS score and stratification variables (treatment centre & no. parents in study). |

The estimand above will answer the question “Is ViPP-SD superior to usual care for behavioural problems in young children, in those that where parents didn’t access alternative interventions and regardless of intervention adherence or Confirmed Neuro-developmental Difficulties, 5 years post randomisation?”

| ***Behavioural Diagnosis estimand attributes***  Estimand attribute | Description |
| --- | --- |
| Population | Participants in the original HSHS RCT |
| Intervention condition(s) | Use of at least 4 of the 6 sessions of VIPP-SD. |
| Variable (outcome) | Standardised total score of Parental Account of Symptoms (PACS) at 5 years post randomisation. |
| Strategies used to handle Intercurrent events | Intervention discontinuation – Treatment Policy  Alternative intervention/s – Treatment Policy  Confirmed Neuro-developmental Difficulties – Principal Stratum |
| Population-level summary measure | Mean difference in PACS score at 5 years since randomisation, between VIPP-SD and usual care adjusting for baseline PACS score and stratification variables (treatment centre & no. parents in study). |

The estimand above will answer the question **“**Is ViPP-SD superior to usual care for behavioural problems in young children that did a Confirmed Neuro-developmental Difficulties during the study period, regardless of adherence and regardless of alternative intervention use, 5 years post randomisation?”

**PRINCIPLE ESTIMAND FOR SECONDARY OUTCOMES**

For all secondary outcomes the same two estimand principles will be used for each outcome. The two estimands will differ by how they handle intercurrent events. The first will hand all intercurrent events as treatment policy, the second estimand will be identical in all attributes but for the intercurrent event of treatment adherence will use a principal stratum approach.

| **Treatment Policy Approach**  Estimand attribute | Description |
| --- | --- |
| Population | Participants involved in the original HSHS trial |
| Intervention | Any use of 6 weeks of the VIPP-SD intervention. |
| Outcome | A Secondary Outcome Variable |
| Strategies for Intercurrent Events | All events – Treatment Policy |
| Population-level summary measure | Mean difference at 5 years post randomisation in parental sensitivity score, adjusted for baseline and stratification variables. |

| **Principle Stratum Approach**  Estimand attribute | Description |
| --- | --- |
| Population | Participants involved in the original HSHS trial |
| Intervention | Using at least 4 of the 6 weeks of the VIPP-SD intervention |
| Outcome | A Secondary Outcome Variable |
| Strategies for Intercurrent Events | Intervention Discontinuation – Principal Stratum  Other events – Treatment Policy |
| Population-level summary measure | Mead difference at 5 years post randomisation in parental sensitivity score, adjusted for baseline and stratification variables. |
